# Influence of Thermal and Mechanical Stimuli on the Behavior of Al-CAU-13 Metal–Organic Framework

**DOI:** 10.3390/nano10091698

**Published:** 2020-08-28

**Authors:** Michael T. Wharmby, Felicitas Niekiel, Jannik Benecke, Steve Waitschat, Helge Reinsch, Dominik Daisenberger, Norbert Stock, Pascal G. Yot

**Affiliations:** 1 Deutsches Elektronen-Synchrotron (DESY), Notkestr. 85, D-22607 Hamburg, Germany; 2 Institut für Anorganische Chemie, Christian Albrechts Universität zu Kiel, Max-Eyth-Straße 2, D-24118 Kiel, Germany; niekiel@ac.uni-kiel.de (F.N.); benecke@ac.uni-kiel.de (J.B.); steve.waitschat@louisenlund.de (S.W.); reinsch@ac.uni-kiel.de (H.R.); stock@ac.uni-kiel.de (N.S.); 3 Diamond Light Source Ltd., Diamond House, Harwell Science & Innovation Campus, Didcot, Oxfordshire OX11 0DE, UK; dominik.daisenberger@diamond.ac.uk; 4 ICGM, University Montpellier, CNRS, ENSCM, F-34095 Montpellier, France

**Keywords:** metal–organic frameworks, Al-CAU-13, thermal stimulus, mechanical stimulus, synchrotron powder X-ray thermodiffraction, in situ high pressure synchrotron powder X-ray diffraction

## Abstract

The response of the metal–organic framework aluminum-1,4-cyclohexanedicarboxylate or Al-CAU-13 (CAU: Christian Albrecht University) to the application of thermal and mechanical stimuli was investigated using synchrotron powder X-ray diffraction (SPXRD). Variable temperature in situ SPXRD data, over the range 80–500 K, revealed a complex evolution of the structure of the water guest containing Al-CAU-13•H_2_O, the dehydration process from ca. 310 to 370 K, and also the evolution of the guest free Al-CAU-13 structure between ca. 370 and 500 K. Rietveld refinement allowed this complexity to be rationalized in the different regions of heating. The Berman thermal Equation of State was determined for the two structures (Al-CAU-13•H_2_O and Al-CAU-13). Diamond anvil cell studies at elevated pressure (from ambient to up to ca. 11 GPa) revealed similarities in the structural responses on application of pressure and temperature. The ability of the pressure medium to penetrate the framework was also found to be important: non-penetrating silicone oil caused pressure induced amorphization, whereas penetrating helium showed no plastic deformation of the structure. Third-order Vinet equations of state were calculated and show Al-CAU-13•H_2_O is a hard compound for a metal–organic framework material. The mechanical response of Al-CAU-13, with tetramethylpyrazine guests replacing water, was also investigated. Although the connectivity of the structure is the same, all the linkers have a linear *e*,*e*-conformation and the structure adopts a more open, wine-rack-like arrangement, which demonstrates negative linear compressibility (NLC) similar to Al-MIL-53 and a significantly softer mechanical response. The origin of this variation in behavior is attributed to the different linker conformation, demonstrating the influence of the S-shaped *a*,*a*-conformation on the response of the framework to external stimuli.

## 1. Introduction

Metal–Organic Frameworks (MOFs) are an important class of coordination polymers with more than 20,000 known structures. The porous nature of this family of compounds as well as their structural diversity has led to a wide range of potential applications being investigated. The properties of these materials which could lead to applications include: gas adsorption/separation [1,2,3,4], catalysis [5,6], drug delivery and biomedicine [7,8], photoluminescence sensors [9,10,11,12,13], magnetism [14,15,16,17], proton conductivity [18,19,20,21], or mechanical energy storage, among others [22,23,24,25,26,27,28,29,30,31,32,33,34,35,36,37,38,39,40]. Prior to its further use for an application, significant effort must be devoted to the characterization of the thermal and mechanical stability of MOFs [41,42]. Flexible MOFs in particular have been intensively investigated [43,44]. MIL-53 (MIL: Materiaux Institut Lavoisier, Versailles, France) in particular has attracted much attention, due to the so-called breathing effect, facilitated by the structure’s wine-rack topology of 1D lozenge-shaped channels [45]. Isoreticular MIL-53 structures have shown a range of different breathing behaviors depending on the nature of: (i) the metal center, for example Al, Cr, Fe, and Ga; and (ii) the organic linkers, including functionalized/unfunctionalized benzene-1,4-dicarboxylates (BDCs) [46,47], naphthalene-2,6-dicarboxylate [48], fumarate [49], and 2,5-thiophendicarboxylate [50]. A review published by Millange and Walton summarizes the numerous studies of the thermal and mechanical properties of flexible MIL-53 and its structure analogs, as well as their phase transitions [51]. A model example of a phase transition in MIL-53 is that between the large pore-form (space group: *Imcm*) and the narrow pore-form (space group: *C2/c*). Interestingly this transition may be induced by: (i) interactions with adsorbed molecules (summarized in reference [51]); (ii) temperature [52,53]; (iii) pressure [28]; and (iv) electrical field [54,55].

**Figure 1:**
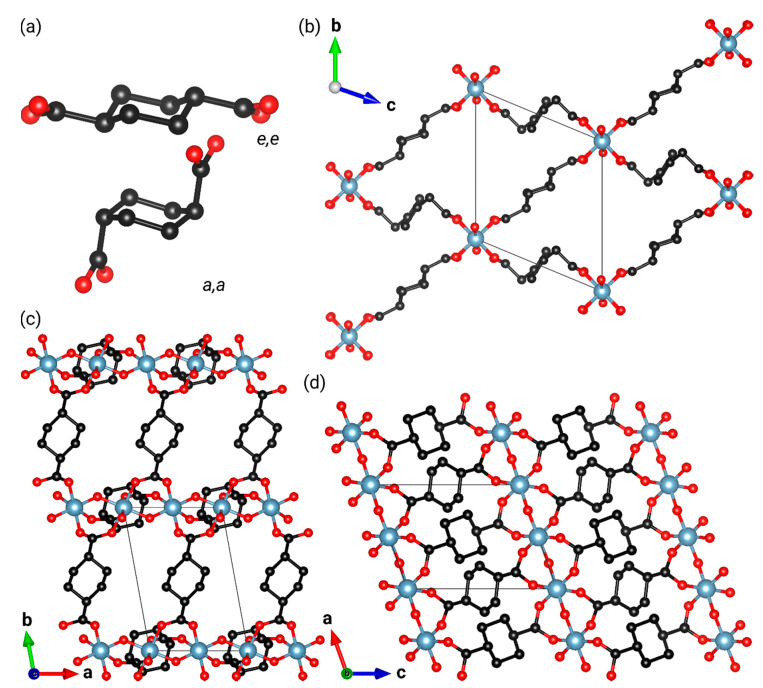
(**a**) The two linker conformations adopted by *trans*-cyclohexan-1,4-dicarboxylate linkers in Al-CAU-13: *e,e*, equatorial,equatorial; *a,a,* axial,axial. Views of the structure of guest-free Al-CAU-13 from Rietveld refinement at 500 K are also shown: (**b**) along the uniaxial channel direction (*a*-axis); and parallel to the *c*- (**c**) and *b*-axes (**d**), showing the connectivity of the linkers and the structures of the chains. N.B. *e,e*-conformation linkers connect the chains in the [011] direction, whereas *a,a*-conformation linkers connect the chains in the [$0\bar{1}1$] direction.

Structural flexibility may also be induced in solids through the use of linker molecules with inherent flexibility. A case in point is the flexible UiO-66 analog, ZrCDC [56], where *trans*-cyclohexan-1,4-dicarboxylate (CDC^2−^) replaces the BDC^2−^ linker molecules. In ZrCDC, flexibility is achieved by a change of the CDC^2−^ linker molecules from an axial,axial (*a*,*a*) to equatorial,equatorial (*e*,*e*) conformation of the carboxylate groups (Figure 1a). A similar linker substitution is also known for the MIL-53 family, with Al-CAU-13 ([Al(OH)(*trans*-CDC)]; CAU: Christian Albrechts Universität) being the first example of this kind of flexibility [57,58] (Figure 1). The compound was first synthesized with H_2_O guest molecules (denoted Al-CAU-13•H_2_O) and crystallizes with a 1:1 ratio of CDC^2−^ linker molecules in the *a*,*a-* and *e*,*e*-conformations. Al-CAU-13 undergoes a breathing transition through conformational changes of the linker, induced by the adsorption of guest molecules [58,59]. Additionally, a computational study has suggested that the guest free form of Al-CAU-13 may show a narrow pore to large pore transition, similar to that known for MIL-53 [60].

We present here the study of the behavior of Al-CAU-13•H_2_O as function of: (i) temperature over the range 80–500 K; and (ii) mechanical pressure up to 11 GPa, using two pressure transmitting media (PTM): silicone oil and helium (for hydrostatic conditions). The mechanical behavior of Al-CAU-13 loaded with tetramethylpyrazine (denoted Al-CAU-13@Tet) was also investigated [59].

## 2. Materials and Methods

### 2.1. Materials Synthesis

The synthesis of Al-CAU-13 was carried out in a PTFE lined steel autoclave with a volume of 37 mL, following the published procedure [57]. All chemicals are commercially available and were used without further purification. *trans*-H_2_CDC (344 mg, 2.0 mmoL) and AlCl_3_•6H_2_O (483 mg 2.0 mmoL) were mixed in a solution of DMF (11.7 mL) and distilled water (2.7 mL). The reactor was sealed and placed in an oven which was heated to 130°C over the course of 1 h. The reaction was kept at this temperature for 12 h, before being cooled to ambient conditions over 1 h. To remove unreacted *trans*-H_2_CDC, the raw product was first treated solvothermally with DMF and then with ethanol before being dried overnight at 120 °C in air. Upon cooling to room temperature, water molecules were adsorbed and a final product of composition [Al(OH)(*trans*-CDC)] •1.5H_2_O was obtained (calculated: C, 39.8; H, 5.85; found: C, 37.7; H, 5.45). The phase purity was further confirmed by powder X-ray diffraction measurements. Al-CAU-13@Tet was prepared by loading tetramethylpyrazine into the structure of Al-CAU-13, as described by Reinsch et al. [59].

### 2.2. Synchrotron X-ray Powder Diffraction

The thermal and mechanical responses of Al-CAU-13 were investigated by variable temperature and in situ high pressure synchrotron powder X-ray diffraction, respectively.

#### 2.2.1. In-Situ Thermodiffractometry

Thermodiffraction data were collected at Swiss-Norwegian Beamline (SNBL, BM01A) at the European Synchrotron Radiation Facility (ESRF, Grenoble, France). The variable temperature diffraction patterns were collected using a PILATUS 2M detector with a sample-detector distance of 343.71 mm and a monochromatic wavelength of 0.70814 Å. Prior to the experiment, the sample-detector distance, wavelength, and detector parameters were calibrated using NIST standard LaB_6_. The temperature was controlled with an Oxford Cryostream 700+ with working temperature range of 80–500 K. Al-CAU-13•H_2_O powder was loaded into a glass capillary of 0.5-mm diameter, which was sealed before mounting on the sample stage. Diffraction data were collected at 1 K intervals from 80 to 500 K, with the cryostream heating at a ramp rate of 2 K min^−1^. Unit-cell parameters were initially determined by first indexing the diffraction patterns at each temperature, using DICVOL6 [61]. Parameters were then refined by LeBail fitting using the Jana 2006 software suite (Institute of Physics, Praha, Czech Republic) [62]. Rietveld refinements of the diffraction patterns at 82, 190, 275, 316, 367, and 500 K were additionally performed, using TOPAS-Academic v.6. [63].

#### 2.2.2. High Pressure In-Situ X-ray Diffraction

High pressure powder X-ray diffraction data were collected at beamline I15, Diamond Light Source (Oxon, UK). Samples were loaded into membrane Diamond Anvil Cells (mDACs) fitted with diamonds with 400-µm culets and stainless-steel gaskets. In addition to the Al-CAU-13•H_2_O or Al-CAU-13@Tet sample, a few crystals of NaCl and KBr were also loaded into the cell as internal pressure standards. Pressure transmitting medium was added to the cells in the final step, either as a drop of silicone oil (AP100, Sigma) or using the gas loading rig available at I15. The cell was mounted on the beamline and diffraction data were collected with a MAR 345 image plate detector, using an X-ray beam size defined by a 70 µm pinhole and at a wavelength of 0.4246 Å (the Sn K-edge). The sample-to-detector distance was determined using NIST standard LaB_6_ and the calibration was performed using DAWN [64]. High pressure diffraction patterns of Al-CAU-13•H_2_O, KBr and NaCl were collected at increments of 2–5 bars of membrane pressure, with the internal pressure of the cell measured using Ruby fluorescence after every third pressure increase. Ruby pressures were determined using the revised ruby pressure scale [65]. Two-dimensional diffraction patterns were integrated using the DAWN suite [64] and the subsequent data were analyzed using TOPAS-Academic v.6. [63] Cell pressures were determined by fitting the unit cell volumes of NaCl and KBr against the reported Vinet equations of state (for both B1 and B2 phases) [66,67]. Ruby pressures were used as fixed points to confirm the evolution of pressure. For pressures up to 4.4 GPa, it was possible to refine the structure of Al-CAU-13•H_2_O by the Rietveld method, using the model reported by Niekiel et al. as a starting model [57]. Only the cell parameters for these refinements are reported, since refinements of the atomic positions in the triclinic structures would not converge due to the weak reflection intensity compared to the background/diffuse scattering. For higher pressures, Rietveld refinement was unsuccessful and Pawley or LeBail fits of the data were performed instead.

## 3. Results and Discussion

### 3.1. In Situ Synchrotron X-ray Thermodiffraction

**Figure 2:**
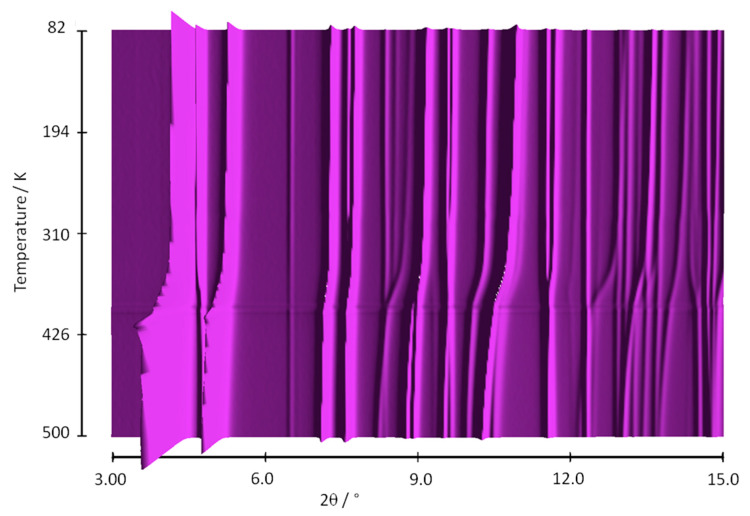
Sequential plot of the diffraction patterns of Al-CAU-13 within the range of temperature 80–500 K. Data measured with X-ray wavelength 0.70814 Å.

Analysis of the thermodiffraction data revealed three distinct regions: the first extending from 80 to 310 K; the second, associated with rapid shifts of the peak positions, from ca. 310 to 370 K; and the third from ca. 370 to 500 K (Figure 2). LeBail fits were performed to refine the unit cell parameters at each temperature point and selected diffraction patterns were fully refined by the Rietveld method, to elucidate the structural changes occurring on thermal treatment.

Diffraction patterns from the first region are similar to those for the Al-CAU-13•H_2_O phase reported by Niekiel et al., while patterns in the third region are similar to those of the empty pore Al-CAU-13 [58]. To further investigate the nature of the structures in the first and third regions, Rietveld refinements were performed on a dataset collected at 80 K, using Al-CAU-13•H_2_O as a model, and on a dataset measured at 500 K, with the empty pore Al-CAU-13 as a structural model (see Figures S1 and S2). The refinements gave good fits to the data, confirming the nature of the phases present (see below). Thus, the middle region shows the effects of the loss of the guest water molecules on Al-CAU-13. Niekiel et al. also reported laboratory thermodiffraction data for the dehydration of CAU-13, [58], which showed the completion of the dehydration process by 370 K, in excellent agreement with the present dataset.

**Figure 3:**
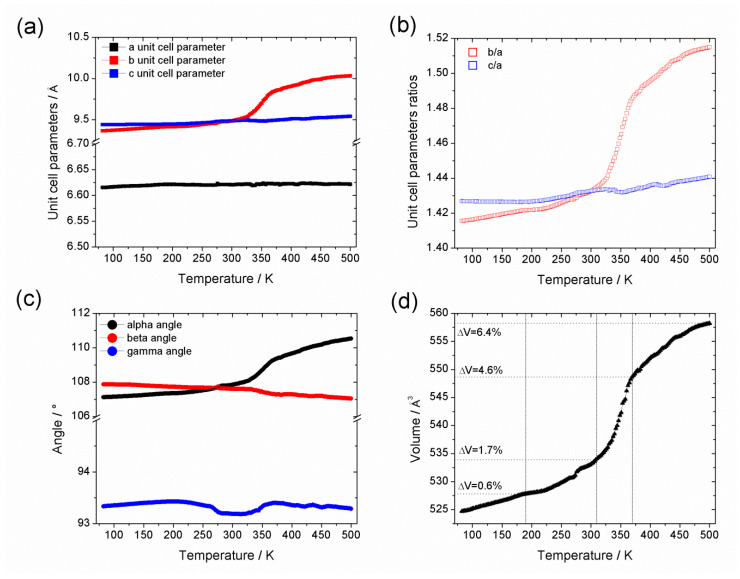
Temperature dependence of: (**a**) the unit cell parameter lengths; (**b**) ratios of unit cell parameter lengths (*b*/*a* and *c*/*a*); (**c**) unit cell parameter angles; and (**d**) unit cell volume of Al-CAU-13 in the temperature range 80–500 K. N.B. region between ca. 310 and 370 K shows the response of the cell to dehydration.

**Figure 4:**
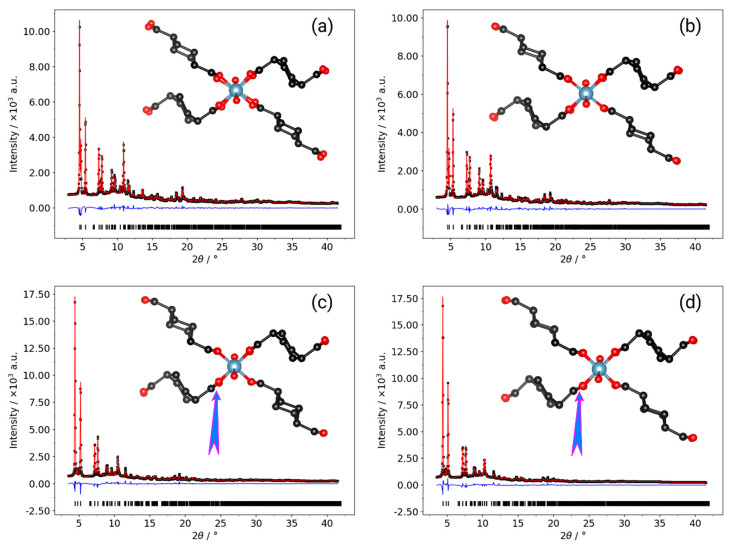
Rietveld plots for the refinements of: Al-CAU-13·H_2_O at 82 K (**a**) and 316 K (**b**); and guest free Al-CAU-13 at 367 K (**c**) and 500 K (**d**). Black crosses are the measured data, red curve is the fitted, and blue curve is the difference plot. Peak positions are shown by ticks underneath. Insets on each plot show a single -Al-(OH)-Al- chain surrounded by four linker molecules taken from the refined structure. Especially in (**c**,**d**), the rotation of the *e*,*e*-linker can clearly be seen. The arrow highlights the backward knee-bend of the *a*,*a*-linker carboxylate groups at 500 K.

The division of the thermodiffraction data into three regions is more clearly shown by the plots of the lattice parameters and cell volume across the temperature range (Figure 3); Rietveld refinements were used to further investigate the structural behavior at selected temperatures (Figure 4, Table S1, and Figures S4–S7). Refinements of the diffraction data at 80 and 316 K using the Al-CAU-13•H_2_O structure give good fits to the data and confirm the presence of guest water molecules, distributed across three partially occupied sites. The refinements confirm that the structure does not undergo any major changes over this temperature range. Plots of the lattice parameters show that the *a*- and *c*-parameters remain approximately constant over the temperature range, whereas the *b*-parameter shows a monotonic increase (ca. 1.5%; Figure 3a). This behavior is unsurprising, since the *b*-direction cuts across the pore, is the least dense and therefore the softest direction in the structure. The *a*-direction is parallel to the inorganic chain axis and is therefore relatively hard, while the *c*-direction is parallel to the *a,a*-linker axis. The cell angles show a more complex behavior, albeit still with small changes (Figure 3c; Δα ~ 0.8%; Δβ ~ −0.3%; Δγ ~ −0.2%). Comparison of the structures refined at 82 and ca. 190 K (see Figure S10) show that the lattice expansion is accommodated by a slight rearrangement of the *e,e*-linker carboxylate groups, which leads to an increase in γ (Δγ ~ 0.1%). Between ca. 190 and 275 K (see Figure S11), a different mode becomes active in the structure, in which the *e,e*-linker rotates around the carboxylate-carboxylate axis, leading to a reduction of γ (Δγ ~ −0.2%). This rotation of the *e,e*-linker is a dominant feature of the structural changes in Al-CAU-13•H_2_O above 190 K. Additionally and as evident from the change in gradient of the *c*/*a* curve (Figure 3b), the cyclohexane ring of the *a,a*-linker flattens slightly and its carboxylate groups twist. This causes an expansion of the *c*-direction (Δ*c* ~ 0.3%) and a consequent increase in the α-angle (Δα ~ 0.3%). From ca. 275 to 316 K, the *e,e*-linkers continue to rotate around the carboxylate axis, but now the *a,a*-linkers also twist in concert around their carboxylate axis (see Figure S12). The lattice adapts through an increase in α (Δα ~ 0.2%), widening the pores slightly, while the other angles remain approximately unchanged. In total, over the temperature range 80–310 K, the structure of Al-CAU-13 increases in volume by approximately 9.5 Å^3^, achieved as described by slight rotations of the linker molecules and small rearrangements of the Al^3+^ coordination sphere, due to movements of the carboxylate groups (see Table S1).

From ca. 310 to 370 K, the structure expands by approximately 14.2 Å^3^, as it responds to the loss of water molecules from the pores (Figure 4 and Table 1). The cyclohexane rings of the *e*,*e*-linkers rotate in the opposite sense around the carboxylate axis. At the same time, there is also a perpendicular rotation of the plane of the ring, which pushes the chains apart and leads to a slight knee bend motion of the carboxylate groups (cf. MIL-53 [45]). At the same time, the *a*,*a*-linker also rotates slightly around an axis parallel to the *a*-direction (rotating about its S-shape), pushing the neighboring chains apart (see Figure S13). Together, these processes cause comparatively large increases in the (soft) *b*-axis (Δ*b* ~ 3.3%) and the α-angle (Δα ~ 1.2%; Figure 3), both of which directly influence the cross-section of the channels. Whereas the loss of guest molecules in MIL-53 causes a significant increase in cell volume, in Al-CAU-13 this is not possible, as noted by Niekiel et al., due to the rigidity of the *a*,*a*-linkers [58]. Moreover, the *S*-shaped geometry of the linker limits the degrees of freedom available to the structure: significant further rotation of the linker would pull the chains back together, rather than further apart.

Rietveld refinements at 367 and 500 K using the structure of guest free Al-CAU-13 gave good fits to the data. The structural changes occurring in this compound are less diverse than for the water guest containing structure at low temperature: all the lattice parameters show monotonic changes over the region (Figure 3). The increase in unit cell volume of approximately 9.9 Å^3^ (Figure 3d) is achieved through increases in the *b*-axis (Δ*b* ~ 2.0%) and the α-angle (Δα ~ 1.2%), leading to a small increase in the volume of the channels (see Table S2). The increases in lattice parameters are mediated through the rotation of the *e*,*e*-linker around the carboxylate axis in the opposite sense to its rotation during dehydration (see Figure S14). The *S*-shaped *a*,*a*-linkers rotate a little further around *a*-axis, causing the carboxylate group to bend back on itself in a knee bending fashion (Figure 4d, inset). Considering the geometry of the carboxylate, it seems that the structure adopted at 500 K is close to the maximum extension available to this mode, and this may account for the decreasing rate of change of the cell volume.

Further insight into the thermal behaviors of Al-CAU-13•H_2_O and guest free Al-CAU-13 can be obtained by determining the parameters of the thermal Equation of State (EoS) (see Figure S15). The Berman EoS was used as it provides a model for the thermal expansion behavior which makes few approximations [68] (see Supplementary Materials, §4.1). A single EoS could not be fitted to the cell volume data for Al-CAU-13•H_2_O (Table 2). The structure expands almost linearly from 80 to 190 K; above 190 K, thermal expansion became non-linear. Considering that, above 190 K, rotation of the *e*,*e*-linkers about their carboxylate axis becomes a common feature of the structural changes occurring, it seems probable this is the reason for non-linear behavior.

Guest free Al-CAU-13 demonstrates a non-linear thermal expansion behavior from 370 to 500 K. Whilst the 0th-order coefficient, α_0_, is fairly similar to the non-linear region of Al-CAU-13•H_2_O, as might be expected, the first-order coefficient, α_1_, has a similar magnitude but opposite sign. As discussed, the rotation of the S-shaped *a*,*a*-linker around the *a*-direction is limited by the geometry of the framework and the carboxylate groups of this linker show backwards knee bending. As the temperature increases, it becomes more difficult for the structure to expand further and thus a negative non-linear behavior occurs.

The EoS for Al-CAU-13 was also determined from a second laboratory dataset, previously reported by Niekiel et al. [58]. In contrast to the synchrotron data, the laboratory dataset showed a more linear thermal behavior, and with significantly smaller magnitudes for α_0_ and α_1_ terms. The difference in the EoS likely reflects the more dynamic nature of the synchrotron experiment, where the temperature was constantly changing, compared to the more static nature of the laboratory experiment (see Figures S16 and S17 and Table S3).

### 3.2. In Situ Synchrotron High Pressure X-ray Diffraction

The structural behavior of Al-CAU-13•H_2_O and Al-CAU-13@Tet (Tet: tetramethylpyrazine guests adsorbed) as a function of mechanical pressure was investigated by high-pressure powder diffraction measurements, using membrane diamond anvil cells (mDACs). Two mDACs containing Al-CAU-13•H_2_O were prepared, one with helium as the pressure transmitting medium and the other with silicone oil. Al-CAU-13@Tet was studied only using silicone oil as the pressure medium (see Supplementary Materials, Figure S3).

**Figure 5:**
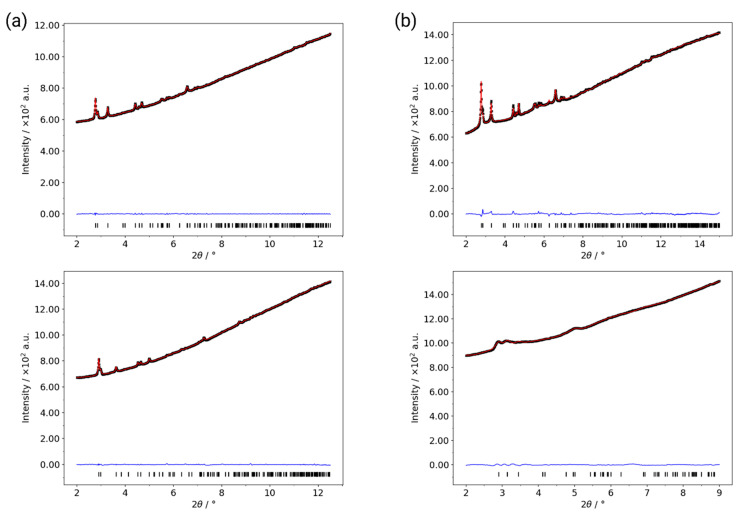
Refinement plots of Al-CAU-13·H_2_O the lowest and highest pressures with (**a**) helium and (**b**) silicone oil as the pressure transmitting medium (PTM). In helium, the lowest pressure (**a**) (**top**) was 0.25 GPa and the highest (**a**) (**bottom**) was 8.09 GPa (see Figures S18 and S19). For silicone oil, the lowest pressure (**b**) (**top**) was 0.42 GPa and the highest (**b**) (**bottom**) was 10.80 GPa (see Figures S20 and S21). The data for the lowest pressure in silicone oil (**b**) (**top**) were fitted by Rietveld refinement; other datasets were fitted by Pawley fits.

The diffraction patterns recorded for the lowest pressures in both helium (Figure 5a) and silicone oil (Figure 5b) scenarios were consistent with the triclinic unit cell reported for Al-CAU-13•H_2_O [57].

**Figure 6:**
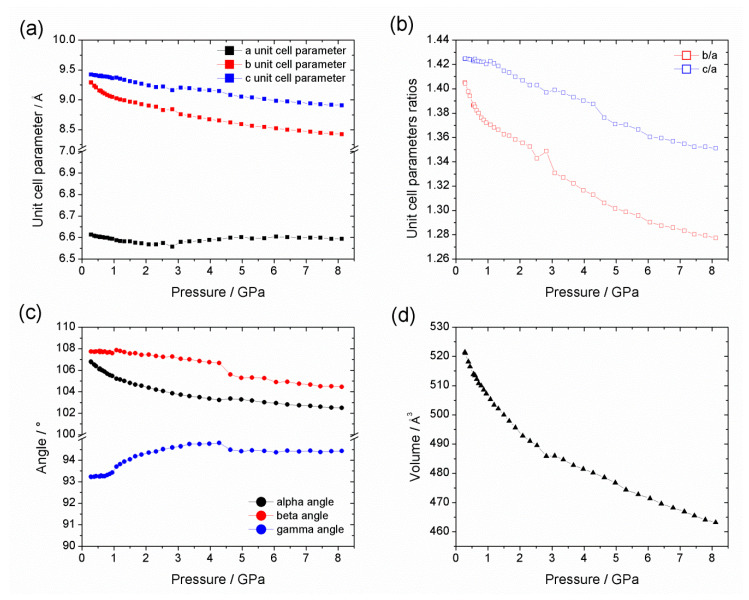
Pressure dependence of: (**a**) the unit cell parameter lengths; (**b**) ratios of the unit cell parameters lengths (*b*/*a* and *c*/*a*), (**c**) unit cell parameter angles; and (**d**) unit cell volume of Al-CAU-13•H_2_O with helium as pressure transmitting medium from ambient pressure up to 8 GPa.

Under compression in helium (Figure 5a) over the range 0.28–8.11 GPa, the structure remained highly crystalline (Figure 6), as indicated by well-defined diffraction peaks, and demonstrated a unit cell volume decrease of approximately −66 Å^3^ (ΔV/V_0_ ~ 13%, Δρ = 0.208 g·cm^−3^; Figure 6d). This compression is achieved primarily by a decrease in the lengths of the *b*- and *c*-direction, while the *a*-axis remains approximately constant. This is a similar behavior to that observed with MIL-53 [32,69], since the relatively incompressible -Al-(OH)-Al- chains are parallel to the *a*-direction in Al-CAU-13. Likewise, in the thermodiffraction study Al-CAU-13, the *a*-direction hardly changed on heating, whereas the *b*- and to a lesser extent *c*-directions showed significant changes.

Although the plot of volume against applied pressure (Figure 6d) shows a single compression process, the variation of the individual lattice parameters indicate that the compression can be subdivided into three stages (Figure 6a,c): the first from 0 to ca. 1.1 GPa; the second from ca. 1.1 to 4 GPa; and the third continuing from ca. 4 GPa to the maximum pressure in the experiment (see Figures S18 and S19).

**Figure 7:**
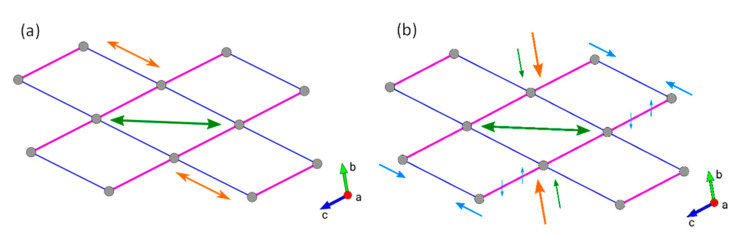
Schematic views of the structural changes occurring in the framework of Al-CAU-13·H_2_O with He (**a**) or silicone oil (**b**) as the PTM. Arrows indicate direction of motion. Green arrows indicate first stage of compression; orange arrows the second stage; and blue arrows the third stage. See text for full details.

In the first stage, the compression is taken up principally by a shortening of the less densely packed *b*-direction and a decrease in the α-angle; the *c*-direction is initially harder to compress than the *b*-direction thanks to rigidity of the linkers with an *a*,*a*-configuration. From a structural point of view, these changes in lattice parameters are accommodated by the lozenge shaped channels adopting a flatter and narrower profile (Figure 7a, green arrow), thus reducing the pore volume. These structural changes are closely related to the structural response to temperature in the thermodiffraction experiment, albeit with a greater magnitude and in the opposite sense (i.e., a pressure increase is equivalent to temperature decrease, whereas in the thermodiffraction experiment the sample was only heated). Above ca. 1.1 GPa, the γ-angle decreases asymptotically towards 94.4°, while the rate of shortening of the *b*-direction decreases. This would increase the distance between chains along the [011] direction (Figure 7a, orange arrow), flattening the channels even further. Both of these processes could be mediated by a knee-bend motion, as observed in the thermodiffraction experiment as well as in the breathing of MIL-53 [45]. The final stage of compression (above 4.3 GPa) involves the simultaneous compression of the *b*- and *c*-directions and gradual decreases in the α- and β-angles in an even fashion. Structurally, this is probably achieved by an isotropic decrease in the size of the channels in the *bc*-plane. The sample was compressed to a maximum pressure of 10.6 GPa but the sample did not show any significant peak broadening. Moreover, on decompression, the sample gave a diffraction pattern very similar to its uncompressed state.

In a second experiment, AP100 silicone oil was also used as PTM to study Al-CAU-13•H_2_O (Figure 5b); this allowed the influence of penetrating and non-penetrating PTM on the compressibility of the solid to be investigated. Two distinct volume decreases were observed over the range 0.42–10.80 GPa. The first is complete by 1.75 GPa and results in a reduction in cell volume of ca. −27 Å^3^ (V/V_0_ ~ 5%, Δρ = 0.078 g·cm^−3^; blue region of Figure 9). Over the entire range of this compression, the sample gave very well-defined diffraction peaks with little broadening (see Figures S20–S22). This compression is accounted for principally by a reduction of the α-angle and a shortening of the *b*-direction larger than that observed with helium. Thus, the effect on the structure (Figure 7b, green arrows) is similar to the first stage of compression with helium as PTM. The larger decrease in the *b*-axis occurs since the silicone oil cannot penetrate the structure and so there is less internal resistance to deformation. The magnitude of the volume decrease over the same pressure range is, however, lower since both the *a*- and *c*-parameters remain largely unchanged in the silicone oil experiment, whereas for helium the *c*-parameter in particular shows a slight decrease. A similar two-stage compression has also been observed with Cu-BTC [23]. In that case, the initial less compressible, hard regime was attributed to the pressure transmitting fluid penetrating the pore space, while a second softer regime indicated the concerted compression of the guest-framework ensemble. In the present case, however, the silicone oil, with which this two stage compression is observed, cannot penetrate the pores of Al-CAU-13•H_2_O as they are extremely narrow, whereas helium, which is small enough to enter the pore space, shows no such two-stage compression. Thus, the two-stage compression of Al-CAU-13•H_2_O with silicone oil has a different origin than that reported for Cu-BTC.

**Figure 8:**
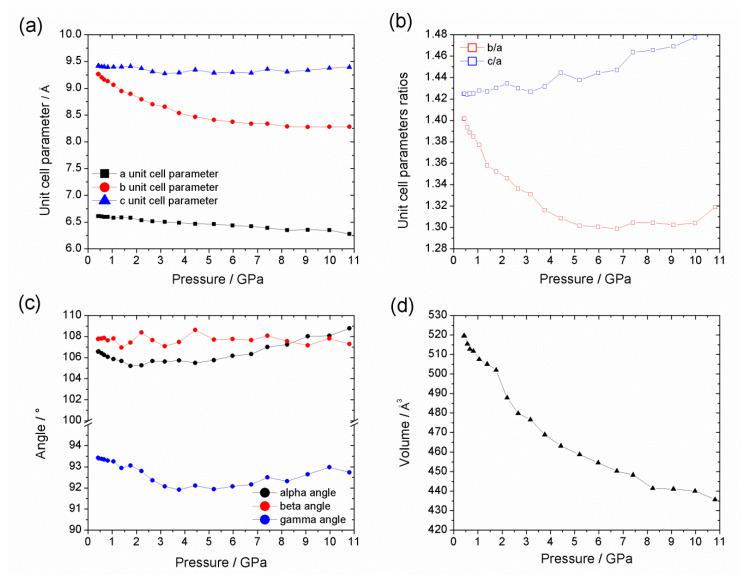
Pressure dependence of: (**a**) the unit cell parameter lengths; (**b**) ratios of the unit cell parameter lengths (*b*/*a* and *c*/*a*); (**c**) unit cell parameter angles; and (**d**) unit cell volume of Al-CAU-13•H_2_O with silicone oil as pressure transmitting medium from ambient pressure up to 11 GPa.

**Figure 9:**
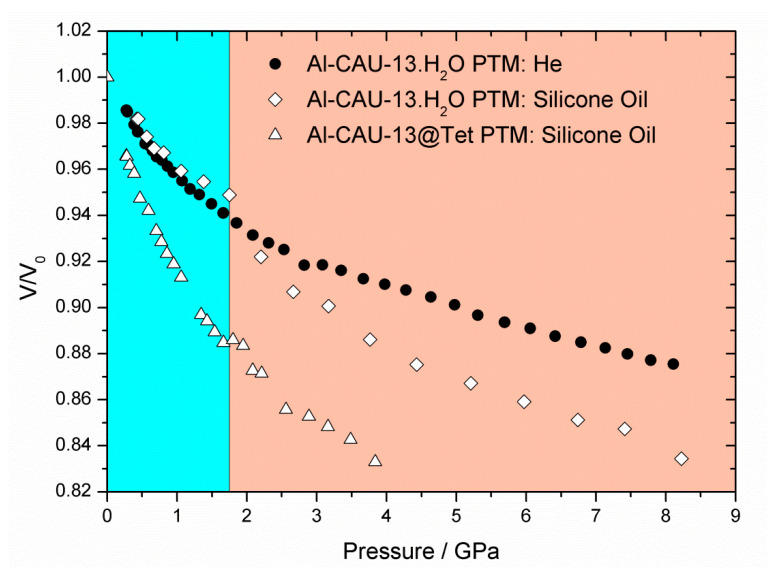
Plot of the fractional volume change of Al-CAU-13•H_2_O as a function of pressure up to ca. 8.5 GPa, with silicone oil (white diamonds) or helium (black circles) as the pressure transmitting media. Compression of Al-CAU-13@Tet in silicone oil (white triangles) shows the dramatically different behavior of the more open framework structure. The blue colored area indicates the first compression of Al-CAU-13•H_2_O in silicone oil while the orange region indicates the second stage of compression.

The second stage compression, from 1.75 GPa (Figure 8b,d) to beyond the end of the measurement (orange region of Figure 9), is associated with Pressure Induced Amorphization (PIA) of the sample (indicated by extreme peak broadening) (see Figure S22). At 8.28 GPa, the unit cell volume had reduced by ca. −87 Å^3^ (V/V_0_ ~ 17%, Δρ = 0.287 g·cm^−3^). Plots of the individual lattice parameters again indicate that the compression can be further subdivided (Figure 8a,c), into two stages. The first continues up to 4 GPa and is associated with decreases in the *a*- and *c*-directions, as well as the γ-angle, which had all remained largely unchanged during the initial compression. Pore space is reduced during these changes presumably through the lozenge-shaped channels becoming flatter without becoming wider (Figure 7b, orange arrows). Above 4 GPa, additional compression is accommodated through an increase in the α-angle concertedly with the shortening of the *b*-direction. This probably decreases the pore space further, through a reduction of the distance between neighboring chains connected by *e*,*e*-linkers; a slight rotation of the *a*,*a*-linkers would probably also be necessary (Figure 7b, blue arrows). Compression of Al-CAU-13•H_2_O to a pressure of 10.80 GPa resulted in extremely broad diffraction peaks, which were impossible to reliably fit. After decompression, the sample showed the same diffraction pattern of broad peaks, indicating that the PIA is irreversible.

The changes occurring in the *a*-direction as part of the second stage of the compression may hint at the origin of the partial amorphization. As this direction is parallel to the -Al-(OH)-Al- chains, it is the hardest and a change in the lattice parameter probably indicates a significant structural change (e.g., bond breaking). That this direction only changes significantly when non-penetrating silicone oil is used as the PTM is also significant. As the silicone oil molecules are unable to penetrate the structure, stresses develop which cannot be compensated by pressure from within the structure/pores. Moreover, under relatively low-pressure conditions the silicone oil no longer provides a hydrostatic compression as it solidifies. This non-hydrostatic compression causes additional (shear) stress on the structure. By contrast, helium is able to penetrate the pore structure, compensating stresses from external pressure and is known to provide hydrostatic compression over a wide pressure range. In the compression of silicalite zeolite with penetrating and non-penetrating PTMs, Haines et al. observed similar PIA only with a non-penetrating PTM [70].

Compression of Al-CAU-13@Tet [59] with silicone oil as the pressure transmitting medium shows a drastically different behavior (Figure 9). In contrast to the other Al-CAU-13 structures discussed, Al-CAU-13@Tet exhibits an orthorhombic crystal system (space group *Imma*), with all cyclohexanedicarboxylate linkers in an *e*,*e*-conformations (see Figures S3, S23, and S24). In this configuration the compound more closely resembles Al-MIL-53. Compression occurs in only one stage (cf. Al-CAU-13•H_2_O in helium), but the compound exhibits slight negative linear compressibility (NLC; Figure 9). Over the range 0−4.19 GPa, the unit cell decreases in volume by −237 Å^3^ (ΔV/V_0_ ~ 19%, Δρ = 0.025 g·cm^−3^). Most of the compression is taken up with a shortening of the *c*-axis (Δ*c* = −18.2%), although there is also a slight compression along the -Al-(OH)-Al- chains (the *a*-axis; Δ*a* = −2.7%). However, over the same range, the *b*-axis elongates very slightly (Δ*b* = 2.1%). This NLC behavior is similar to that observed for other MIL-53 compounds and is caused by the wine-rack-like connectivity of the framework [32]. Serra-Crespo et al. demonstrated that compression of MIL-53 beyond 3 GPa leads to a second phase of compression, in which all three lattice parameters decrease with pressure [69]. The tetramethylpyrazine guest molecules provide additional resistance to compression along the *b*-direction, thus it is probable that this second phase of compression begins at higher pressures with Al-CAU-13@Tet.

**Figure 10:**
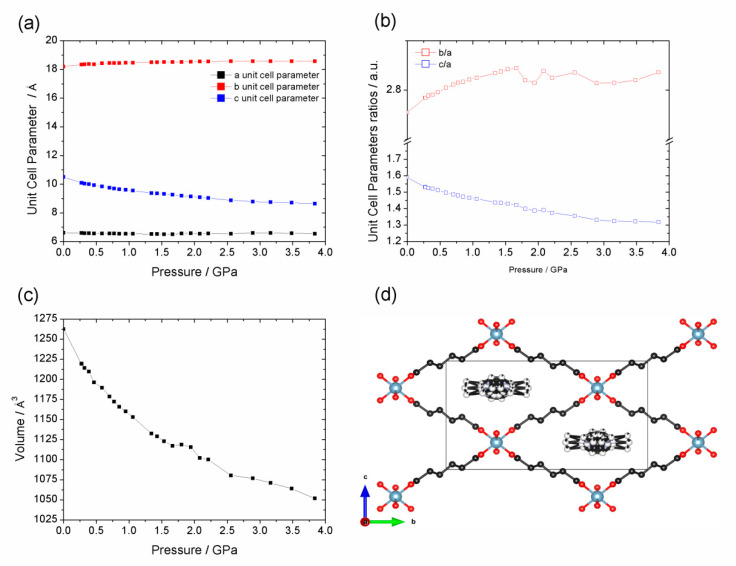
Pressure dependence of: (**a**) the unit cell parameters lengths; (**b**) unit cell parameters (*b*/*a* and *c*/*a*) ratios; and (**c**) unit cell volume of Al-CAU-13@Tet with silicone oil from ambient pressure up to 4 GPa. (**d**) The structure of Al-CAU-13@Tet viewed along the *a*-axis, as reported by Reinsch et al. [59].

The bulk moduli for the compression of Al-CAU-13•H_2_O in helium, the two compression regimes of Al-CAU-13•H_2_O in silicone oil and also Al-CAU-13@Tet in silicone oil (Figure 10) were determined from the third-order Vinet EoS [71], from fits of the volume vs. pressure curves using the program EoSFit7 (Table 3; see Supplementary Materials, §4.2). V_0_, K_0_, and K’ values were all fitted; fits weighted using σ(p) values were found to give the best fits to the data (see Figures S25–S27).

The bulk moduli calculated for Al-CAU-13•H_2_O in helium (K_0_ = 25.08 GPa) and for the initial compression in silicone oil (K_0_ = 24.55 GPa; blue region of Figure 9) are very similar, indicating that the compression is mediated by similar structural processes in both cases, regardless of the pressure medium. The bulk moduli calculated are surprisingly high in comparison to other MOFs (cf. ZIF-8: K_0_ = 6.5 GPa [23]; MOF-5: K_0_ = 16.52 GPa [72]; UiO-66: 12–26 GPa [73]) and in fact more similar to soft inorganic compounds (cf. NaCl: K0 = 23.83 GPa [66]). This leads to the classification of CAU-13 as a hard MOF and is a direct consequence of the *a,a*-conformation of half of the linkers.

In contrast, in the second phase of compression of Al-CAU-13•H_2_O in silicone oil (K_0_ = 8.93 GPa; orange region of Figure 9), the compound shows a greater compressibility, as evidenced by the drastically smaller bulk modulus. This indicates a very different structural process is occurring, consistent with the PIA observed in the diffraction data and shows that when using non-penetrating PTMs, Al-CAU-13•H_2_O readily and irreversibly undergoes plastic deformation and amorphizes. This is completely analogous to the report of Haines et al. on silicalite zeolites [70]. It should also be noted that the K’ of the second phase of compression is smaller than the first phase (see Figures S26a and S14b), indicating a higher resistance to the plastic deformation of the framework. This again is consistent with the observation that changes in the direction parallel to the otherwise hard and dense -Al-(OH)-Al- mark the start of this compressive regime.

The bulk modulus calculated for the compression of Al-CAU-13@Tet is significantly smaller than that of Al-CAU-13•H_2_O (K_0_ = 6.19 GPa), demonstrating the highly compressible nature of the compound (Supp. Mater. Figure S27). The more open structure of the pore space and the less rigid *e*,*e*-conformation of all the linkers (cf. 50:50 *a*,*a*:*e*,*e* in the other Al-CAU-13 structures) allows the compound to deform more easily. Indeed, in this configuration, the framework (which is itself chemically identical to Al-CAU-13•H_2_O) has a mechanical response similar to other MOFs. For example, the bulk modulus of Al-CAU-13@Tet is similar to that reported for Al-MIL-53 (K_0_ = 7.4 GPa for NLC region [69]), confirming the mechanical as well as structural similarity of the compounds. The slightly smaller bulk modulus of Al-CAU-13@Tet (i.e., more compressible) compared to Al-MIL-53 could be due to the more flexible nature of the aliphatic cyclohexane rings in Al-CAU-13.

## 4. Conclusions

The structural response of the metal–organic framework (MOF) Al-CAU-13 to the application of thermal and mechanical stimuli was investigated and compared. Through thermodiffraction experiments, Al-CAU-13•H_2_O was shown to undergo a 1.9% volume increase from 80 to ca. 310 K. Rietveld refinement was used to understand the structural changes occurring and to rationalize the changes in lattice parameters. The structure of Al-CAU-13•H_2_O adapts to the increasing temperature differently in different temperature regions: at temperatures below 190 K through rearrangements of the carboxylate groups; at higher temperatures through rotations of the *e*,*e*-linker cyclohexane ring around the carboxylate axis. This sequence of changes of structural response explains why it was not possible to fit a single Berman thermal Equation of State (EoS) across this temperature range.

Between 310 and ca. 370 K, Al-CAU-13 loses guest water molecules from the pores, which causes the structure to expand by ca. 14 Å^3^. This was found to be in good agreement with previous literature reports [58]. The transition from hydrated to dehydrated structure is meditated principally by rotations of the *e*,*e*-linkers about the carboxylate axis and also a slight rotation of the *a*,*a*-linker around its S-profile. Interesting, the rotation in the *e*,*e*-linkers is in the opposite sense to that which facilitates the expansion of the structure at low temperatures.

Above 370 K, the dehydrated structure continues to expand, increasing the cell volume by 1.8% up to 500 K. Expansion in this region is dominated by rotations of the *a*,*a*-linker about its S-profile, which leads to a slight backward knee bend of the carboxylate groups at the highest temperatures. Al-CAU-13 is unable to demonstrate the dramatic breathing of the related Al-MIL-53 due to the conformation of the *a*,*a*-linkers: further rotation of the S-shaped linkers, much beyond that observed in the 500 K structure, would likely pull the chains back together, which would result in negative thermal expansion (NTE). Clearly, the linear shape of the linkers in Al-MIL-53 is crucial to enable the wine-rack like motion which is the origin of the breathing.

The *b*-axis and the α-angle, which define the profile of the pore channels, showed the largest and most consistent changes across the whole temperature range of the thermodiffraction study. Similarly, in the high-pressure study, the *b*-axis of Al-CAU-13•H_2_O showed the largest shortening with either helium or silicone oil as the pressure transmitting medium (PTM). With helium, a single compressive regime was observed across the entire pressure range studied (ambient to at least 8 GPa), whereas, with silicone oil, a two-stage compression occurs (ambient to 1.75 GPa and 1.75 GPa to at least 11 GPa), resulting in pressure induced amorphization (PIA); such PIA has previously evidenced with penetrating and non-penetrating PTMs in zeolites [70]. By contrast, compression of the compound Al-CAU-13@Tet (tetramethylpyrazine guests adsorbed in place of water molecules) in silicone oil demonstrated a significantly softer behavior than Al-CAU-13•H_2_O, even in comparison with the PIA regime, and showed no evidence of amorphization. This is attributed to the more open network of the structure, which is able to flex in a wine-rack motion (cf. MIL-53). This similarity of the mechanical response between Al-CAU-13@Tet and MIL-53 is confirmed by the observation of a small Negative Linear Compressibility (NLC) behavior, attributed to this wine-rack topology.

A third-order Vinet EoS was fitted to each compression. The bulk moduli, K_0_, determined for the first stage of compression of Al-CAU-13•H_2_O in silicone oil and in helium are approximately equal (ca. 25 GPa), whereas the K_0_ for the second stage compression in silicone oil is significantly lower (K_0_ ~ 8.9 GPa); the mechanical response of Al-CAU-13@Tet in silicone oil is softer still (K_0_ ~ 6.2 GPa). In comparison to inorganic solids, Al-CAU-13•H_2_O has a similar mechanical response to soft alkali halide salts, e.g., NaCl, K_0_ = 23.83 GPa [66] (the PIA region is taken to be unrepresentative of the mechanical properties of the compound). However in comparison to other MOFs, a K_0_ of ca. 25 GPa represents a hard, relatively incompressible compound (ZIF 8: K_0_ = 6.5 GPa [23]; UiO-66: 12–26 GPa [73]). Moreover, the bulk modulus of Al-CAU-13@Tet indicates a very soft (even for a MOF) mechanical response.

To understand the origins of these starkly differing hard/soft behaviors demonstrated by the same framework, it is instructive to consider the conformation of the linkers in the structure and also the nature of the guests in the pores. As a penetrating PTM, helium atoms would be expected to occupy the pore space, whereas molecules of silicone oil—a non-penetrating PTM—cannot. That Al-CAU-13•H_2_O only undergoes PIA under compression in silicone oil provides confirmation for this: the absence of an opposing pressure from the pores would reduce the resistance of the compound to plastic deformation. As compression of Al-CAU-13•H_2_O in both PTMs yields the same bulk moduli, the presence or absence of guests has no influence on the mechanical properties observed, and it is therefore the mechanical properties of the framework itself that are determined. Assuming this also to be the case for the compression of Al-CAU-13@Tet in silicone oil, it is clear that the conformation of the linker is the defining feature of the mechanical properties of Al-CAU-13. In the case of Al-CAU-13•H_2_O, the 50:50 *a*,*a*:*e*,*e* ratio of linker conformations leads to a significantly harder framework, which is mechanically unable to flex and in which extension is limited by the S-shape of the *a*,*a* linker (as observed in the thermodiffraction experiment). Moreover this *a*,*a* conformation could lead to additional weak dispersive interactions between the linker and other parts of the framework, which have previously been shown to have a decisive role on structural behavior [36]. By contrast the open, wine-rack-like network adopted by Al-CAU-13@Tet allows the structure to flex in a similar manner to MIL-53 (i.e., a knee-bend motion about the carboxylate groups) [45]. The less dense arrangement of the linker also means that fewer dispersive interactions can develop and hence there are fewer constraints on motions of the linker.

Regarding the reversibility of the effect of thermal and mechanical stimuli on Al-CAU-13, we observe that under mechanical stimulus and in the absence of PIA, the structural changes were found on decompression to be reversible. PIA, by its very nature is, however, irreversible, and its onset therefore represents an upper limit of the mechanical stress that the framework can endure without permanent deformation. In the case of thermal stimulus, although this was not investigated in the present work, Niekiel et al. [58] demonstrated that the framework breathing is reversible on cooling in the presence of atmospheric moisture. We therefore suspect that, as the structural changes presented herein do not require bond breaking or formation, the thermal structural changes will also be fully reversible. Obviously, under vacuum, the compound would be unable to reabsorb water molecules into the pore space. Further experiments would be required to determine the structure adopted in the absence of guest molecules at low temperatures, although, on the basis of other MIL-53 structures [45], the more open framework structure might be expected to be stable, due to the absence of guest-framework interactions.

Clearly, MOFs made with the cyclohexanedicarboxylic acid linker can flex in the same way as their counterparts made with terephthalic acid. The thermal and mechanical studies of Al-CAU-13•H_2_O show how inclusion of linkers with an S-shaped *a*,*a*-conformation, rather than a linear *e*,*e*-conformation, can influence the thermal and mechanical response of the framework. By selectively including these S-shaped linkers, it may be possible to control whether unusual structural responses (e.g., NLC and NTE) manifest themselves in a framework and what magnitude they have.

## Figures and Tables

**Table 1:** Crystal phase, space group (s.g.), temperature T (K) at which the structure was determined, formula unit Z, and unit cell parameters. * The high GoF values, even with visually good fits and excellent R_wp_/R_Bragg_, are caused by the counting statistics of the detector. Photon counting detectors such as the Pilatus used here regularly give these large GoF values and therefore this is not a negative indicator for the quality of the fit.

	Al-CAU-13·H_2_O		Al-CAU-13	
Formula	[Al(OH)(C_8_H_10_O_4_)] 1.47H_2_O	[Al(OH)(C_8_H_10_O_4_)] 1.29H_2_O	[Al(OH)(C_8_H_10_O_4_)]	
Temp./K	82	316	367	500
Space Group	$P\bar{1}$	$P\bar{1}$	$P\bar{1}$	$P\bar{1}$
Z	2	2	2	2
a/Å	6.6177(4)	6.6197(6)	6.6252(8)	6.6207(12)
b/Å	9.3651(7)	9.5103(10)	9.8233(15)	10.027(2)
c/Å	9.4371(6)	9.4896(8)	9.4899(11)	9.5375(15)
α/°	107.097(4)	107.980(6)	109.229(8)	110.492(10)
β/°	107.915(6)	107.636(10)	107.355(12)	107.054(18)
γ/°	93.336(3)	93.161(9)	93.407(10)	93.335(13)
V/Å^3^	524.76(7)	534.21(10)	548.03(14)	557.78(19)
R_wp_	2.77	3.23	3.62	3.33
R_Bragg_	2.55	2.21	3.01	1.87
GoF *	27.308	29.470	34.287	31.875

**Table 2:** Parameters (V_0_, α_0_ and α_1_) of the Berman thermal equations of state obtained for water guest containing Al-CAU-13•H_2_O in the temperature ranges 84–190 K and 190–316 K and for guest free Al-CAU-13 in the temperature range 378–500 K (see Supplementary Materials §4.1 for Berman EoS).

	Temperature Range (K)	V_0_/Å^3^	α_0_/× 10^−5^ K^−1^	α_1_/× 10^−8^ K^−2^
Al-CAU-13·H_2_O	84–190	524.7s(1) at 84 K	5.1(1)	9.8(1)
	190–316	533.1(1) at 298 K	15.7(3)	118.1(1)
Al-CAU-13	378–500	549.8(1) at 380 K	20.5(3)	−126.3(1)

**Table 3:** Parameters for the Vinet equation of state [71] of Al-CAU-13•H_2_O and Al-CAU-13@Tet. For Al-CAU-13•H_2_O in silicone oil, pressures above 7.41 GPa were excluded from the fits due to the large uncertainty on the volume (see Figures S25–S27; Vinet equation, §4.2).

	Pressure Range (GPa)	V_0_/Å^3^	K_0_/GPa	K’
Al-CAU-13·H_2_O (He)	0.28–8.11	521.16	25.08	17.49
Al-CAU-13·H_2_O (Sil. Oil)	0.42–1.75	525.64	24.55	17.49
	1.75–7.41	547.49	8.93	14.01
Al-CAU-13@Tet (Sil. Oil)	0.27–3.84	1262.54	6.19	13.85

## Supplementary Materials

The following are available online at https://www.mdpi.com/2079-4991/10/9/1698/s1. CCDC 2017417–2017422 contain the supplementary crystallographic data for this paper. These data can be obtained free of charge from The Cambridge Crystallographic Data Centre via www.ccdc.cam.ac.uk/structures. Figure S1: Structure of Al-CAU-13•H_2_O at 82 K from Rietveld refinement, viewed along the *a* axis; Figure S2: Structure of Al-CAU-13 at 500 K from Rietveld refinement, viewed along the *a* axis; Figure S3. Structure of Al-CAU-13@Tet from Reinsch et al. [1], viewed along the *a* axis.; Table S1: Summary of Rietveld refinement results for water-guest containing Al-CAU-13 at 82 K, 189 K, 275 K and 316 K.; Figure S4: Rietveld plot for the final cycle of refinement of water-guest containing Al-CAU-13 at 82 K. Left: full range of refinement; Right: fit over the 2θ range 11° to 42°; Figure S5: Rietveld plot for the final cycle of refinement of water-guest containing Al-CAU-13 at 189 K. Left: full range of refinement; Right: fit over the 2θ range 11° to 42°; Figure S6: Rietveld plot for the final cycle of refinement of water-guest containing Al-CAU-13 at 275 K. Left: full range of refinement; Right: fit over the 2θ range 11° to 42°; Figure S7: Rietveld plot for the final cycle of refinement of water-guest containing Al-CAU-13 at 316 K. Left: full range of refinement; Right: fit over the 2θ range 11° to 42°; Table S2: Summary of Rietveld refinement results for guest-free Al-CAU-13 at 367 K and 500 K; Figure S8: Rietveld plot for the final cycle of refinement of water-guest containing Al-CAU-13 at 367 K. Left: full range of refinement; Right: fit over the 2θ range 11° to 42°; Figure S9: Rietveld plot for the final cycle of refinement of water-guest containing Al-CAU-13 at 500 K. Left: full range of refinement; Right: fit over the 2θ range 11° to 42°; Figure S10: Structures of Al-CAU-13 at 82 K (blue) and 190 K (red) overlaid, showing differences between structures. Guest water molecules not included for clarity; Figure S11: Structures of Al-CAU-13at 190 K (blue) and 275 K (red) overlaid, showing differences between structures. Guest water molecules not included for clarity; Figure S12: Structures of Al-CAU-13 at 275 K (blue) and 316 K (red) overlaid, showing differences between structures. Guest water molecules not included for clarity; Figure S13: Structures of Al-CAU-13 at 316 K (blue) and 375 K (red) overlaid, showing changes in the structure occurring on loss of guest water molecules. Guest water molecules not included for clarity; Figure S14: Structures of Al-CAU-13 at 367 K (blue) and 500 K (red) overlaid, showing differences between structures; Figure S15: Evolution of the unit cell volume for Al-CAU-13 as a function of temperature for the three domains: a) low temperature linear (84–190 K), b) low temperature non-linear (190–316 K) and c) high temperature (367–500 K). Filled circles are the experimental data; determined Berman EoS plotted as a solid line; Figure S16: Comparison of unit cell volume of Al-CAU-13 over the temperature range 310 to 500 K determined from synchrotron (this study) and laboratory (data reported by Niekiel et al. [4]) thermodiffraction data. Niekiel et al. provide two sets of cell volume data: the first were obtained from an as-prepared sample of Al-CAU-13•H_2_O; the second was obtained from the same sample after it had cooled from 500 K back to 303 K; Figure S17: Evolution of the unit cell volume of Al-CAU-13 as a function of the temperature during the first and second heating experiments. The first experiment is split into two regions: (a) 305–353 K and (b) 383–473 K, with differing degrees of non-linear behavior. The first of these represents the dehydration of the compound. The second heating (c) could be fitted with a single curve over the entire region (353–673 K). Filled circles are the experimental data; determined Berman EoS plotted as a solid line; Table S3: Parameters of the thermal Equation of State determined for the three regions plotted in Figure S17; Figure S18: Plot for the Pawley fit of Al-CAU-13•H_2_O at a pressure of 0.26 GPa with helium as the pressure medium (R_wp_ = 0.16 %, GoF = 0.0526); Figure S19: Plot for the Pawley fit of Al-CAU-13•H_2_O at a pressure of 8.09 GPa with helium as the pressure medium (R_wp_ = 0.19 %, GoF = 0.0641); Figure S20: Rietveld plot for the refinement of of Al-CAU-13•H_2_O at a pressure of 0.42 GPa with silicone oil as the pressure medium (R_wp_ = 0.47 %, GoF = 0.1518); Figure S21: Rietveld plot for the refinement of of Al-CAU-13•H_2_O at a pressure of 1.75 GPa with silicone oil as the pressure medium (R_wp_ = 0.35 %, GoF = 0.1376); Figure S22: Plot for the Pawley fit of Al-CAU-13•H_2_O at a pressure of 10.80 GPa with silicone oil as the pressure medium (R_wp_ = 0.23 %, GoF = 0.0799). N.B. A smaller 2θ range (compared to the lower pressure measurements) was used for the refinement to ensure it remained stable; Figure S23: Plot for the Pawley fit of Al-CAU-13@Tet at a pressure of 0.27 GPa with silicone oil as the pressure medium (R_wp_ = 0.56 %, GoF = 0.2193); Figure S24: Plot for the Pawley fit of Al-CAU-13@Tet at a pressure of 4.19 GPa with silicone oil as the pressure medium (R_wp_ = 0.68 %, GoF = 0.2569); Figure S25: Evolution of the unit cell volume for the Al-CAU-13•H_2_O in He over the pressure range 0.28-8.11 GPa (points) with the curve fit for the Vinet EoS; Figure S26: Evolution of the unit cell volume for the Al-CAU-13•H_2_O in silicone oil over the pressure ranges (a) 0.42–1.75 GPa and (b) 1.75–7.41 GPa (points). The curve is the fit for the Vinet EoS; Figure S27: Evolution of the unit cell volume for the Al-CAU-13@Tet in silicone oil over the pressure range 0.27–3.84 GPa (points) with the curve fit for the Vinet EoS.
