# Supplementary material for: Influence of Thermal and Mechanical Stimuli on the Behavior of Al-CAU-13 Metal–Organic Framework
Source: Nanomaterials (Basel). 2020 Aug 28;10(9):1698. doi: 10.3390/nano10091698 (PMC7557782; doi:10.3390/nano10091698)
Supplement: Supplementary file 1 [file nanomaterials-10-01698-s001.pdf]

nanomaterials

Supplementary Materials

Influence of Thermal and Mechanical Stimuli on the Behavior of Al-CAU-13 Metal–Organic Framework

Michael T. Wharmby [1,*], Felicitas Niekiel [2], Jannik Benecke [2], Steve Waitschat [2], Helge Reinsch [2], Dominik Daisenberger [3], Norbert Stock [2] and Pascal G. Yot [4,*]

1 Deutsches Elektronen-Synchrotron (DESY), Notkestr. 85, D-22607 Hamburg, Germany
2 Institut für Anorganische Chemie, Christian Albrechts Universität zu Kiel, Max-Eyth-Straße 2, D-24118 Kiel, Germany; niekiel@ac.uni-kiel.de (F.N.); benecke@ac.uni-kiel.de (J.B.); steve.waitschat@louisenlund.de (S.W.); reinsch@ac.uni-kiel.de (H.R.); stock@ac.uni-kiel.de (N.S.)
3 Diamond Light Source Ltd., Diamond House, Harwell Science & Innovation Campus, Didcot, OX11 0DE Oxfordshire, UK; dominik.daisenberger@diamond.ac.uk
4 ICGM, Univ. Montpellier, CNRS, ENSCM, F-34095 Montpellier, France
* Correspondence: mt@wharmby.org.uk (M.T.W.); pascal.yot@umontpellier.fr (P.G.Y.); Tel.: +33-467-143-294 (P.G.Y.)

1. *Structures of the Phases of Al-CAU-13*

Three phases have been investigated as part of this study:

- water-guest containing Al-CAU-13 (Al-CAU-13•H_2O): [Al(OH)($C_8H_{10}O_4$)]•xH_2O
- guest free Al-CAU-13 (Al-CAU-13): [Al(OH)($C_8H_{10}O_4$)]
- tetramethylpyrazine adsorbed Al-CAU-13 (Al-CAU-13@Tet): [Al(OH)($C_8H_{10}O_4$)]•x($C_4H_4N_2$)

Representative structures of these compounds are given in figures S1, S2 and S3, respectively. All three figures show the same atoms. Guest molecules are only included in the channels within the unit cell.

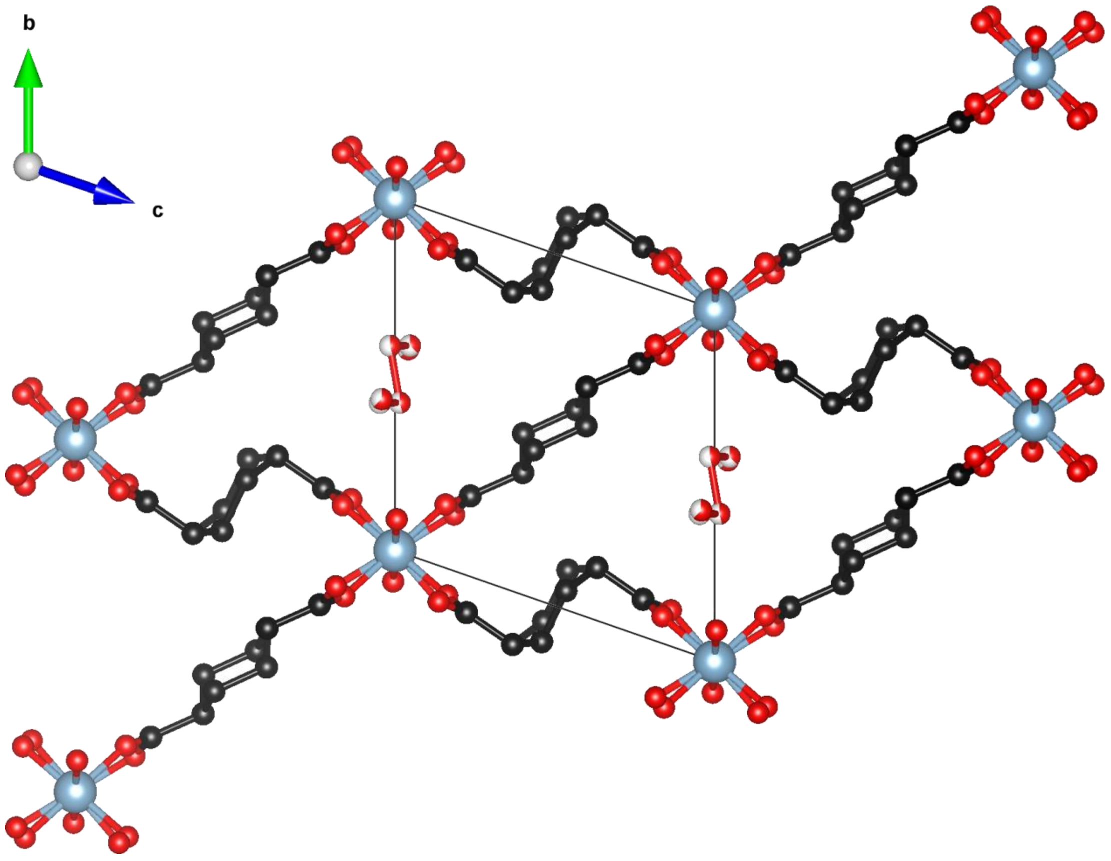

Figure S1. Structure of Al-CAU-13•H_2O at 82 K from Rietveld refinement, viewed along the *a* axis.

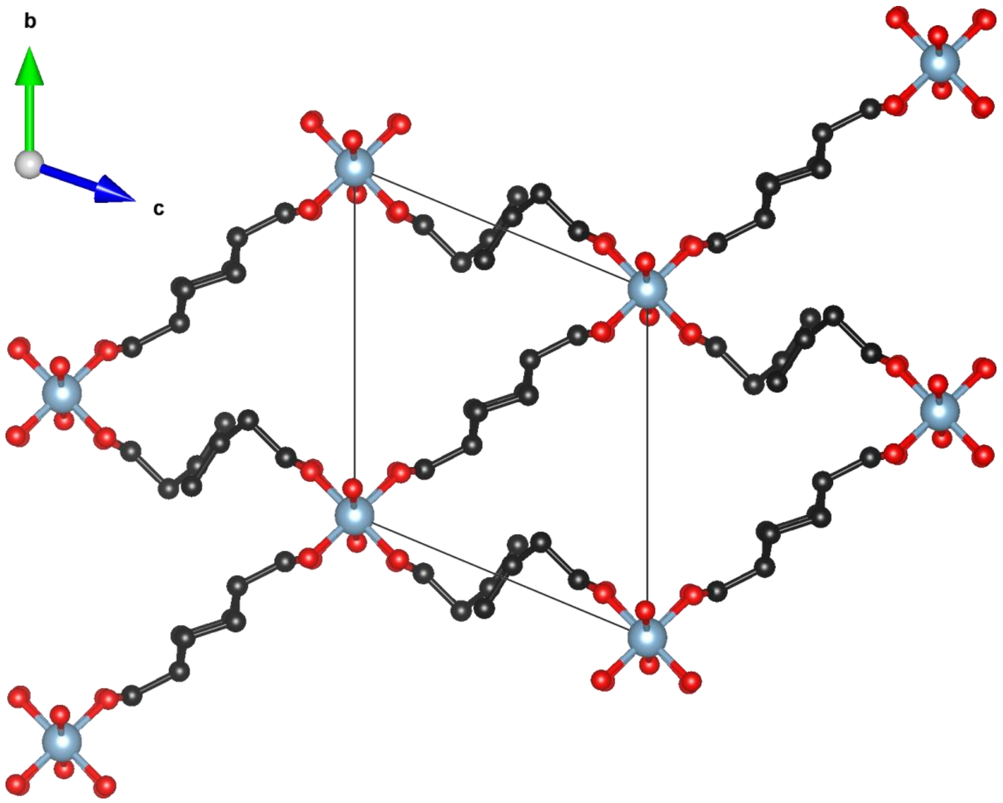

Figure S2. Structure of Al-CAU-13 at 500 K from Rietveld refinement, viewed along the *a* axis.

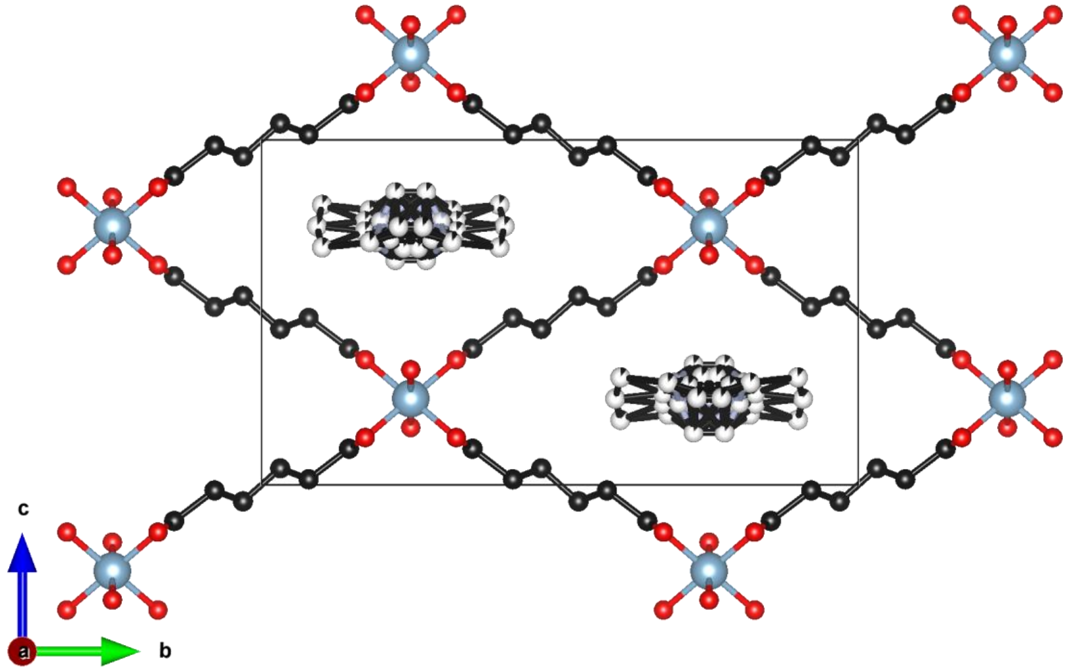

Figure S3. Structure of Al-CAU-13@Tet from Reinsch *et al.*[1], viewed along the *a* axis.

2. *In situ Synchrotron X-ray Thermodiffraction*

Table S1. Summary of Rietveld refinement results for water-guest containing Al-CAU-13 at 82 K, 189 K, 275 K and 316 K.

Temp. / K	82	189	275	316
Formula	$[Al(OH)(C_8H_{10}O_4)]\bullet$ $1.47H_2O$	$[Al(OH)(C_8H_{10}O_4)]\bullet$ $1.42H_2O$	$[Al(OH)(C_8H_{10}O_4)]\bullet$ $1.45H_2O$	$[Al(OH)(C_8H_{10}O_4)]\bullet$ $1.29H_2O$
Space Group	$P\bar{1}$	$P\bar{1}$	$P\bar{1}$	$P\bar{1}$
Z	2	2	2	2
a / Å	6.6177(4)	6.6227(5)	6.6182(5)	6.6197(6)
b / Å	9.3651(7)	9.4145(7)	9.4513(9)	9.5103(10)
c / Å	9.4371(6)	9.4451(6)	9.4752(7)	9.4896(8)
α / °	107.097(4)	107.316(5)	107.691(5)	107.980(6)
β / °	107.915(6)	107.788(7)	107.694(8)	107.636(10)
γ / °	93.336(3)	93.445(6)	93.225(7)	93.161(9)
V / Å^3	524.76(7)	527.89(8)	530.68(8)	534.21(10)
R_{wp} / %	2.77	3.00	3.07	3.23
R_{Bragg} / %	2.55	2.36	2.06	2.21
GoF*	27.308	28.240	28.189	29.470

* The high GoF values, even with visually good fits and excellent R_{wp}/R_{Bragg}, are caused by the counting statistics of the detector. Photon counting detectors such as the Pilatus used here regularly give these large GoF values and therefore this is not a negative indicator for the quality of the fit

Figures S4 to S7 show Rietveld plots for the refinements shown in Table S1. In the plots, black crosses show measured data, the red line is the calculated diffraction pattern and the blue line is difference between measured and calculated. Expected peak positions are indicated with vertical ticks at the bottom of the plot.

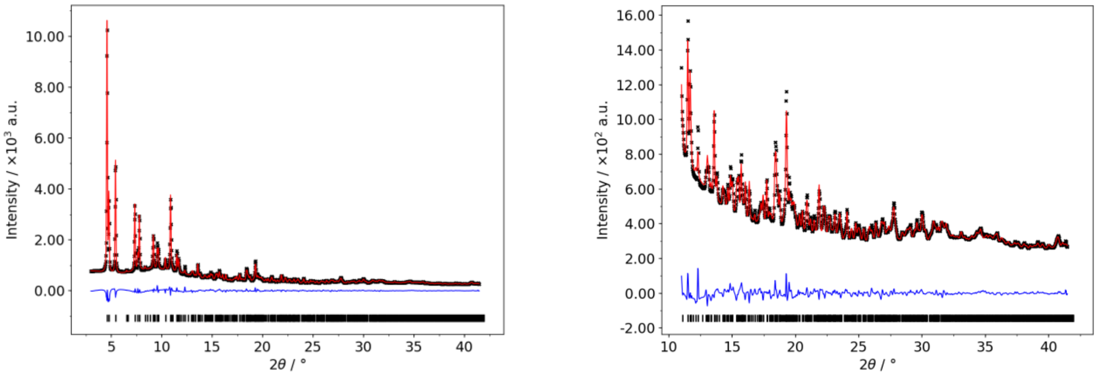

Figure S4. Rietveld plot for the final cycle of refinement of water-guest containing Al-CAU-13 at 82 K. Left: full range of refinement; Right: fit over the 2θ range 11° to 42°.

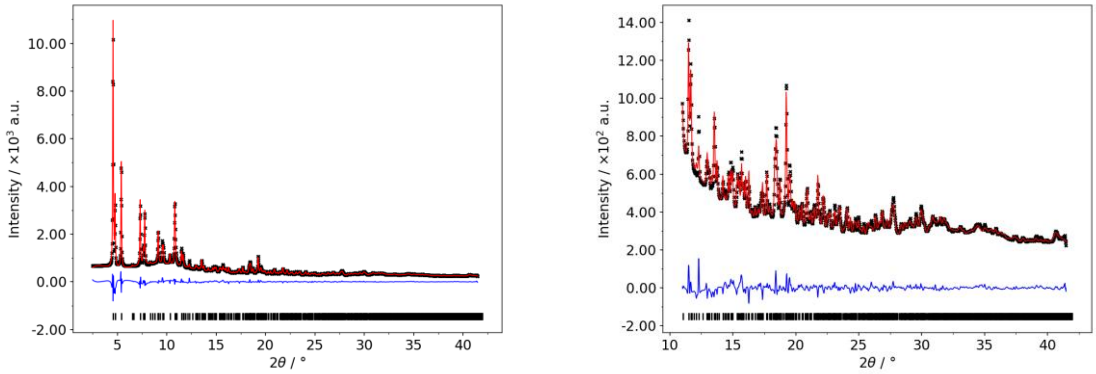

Figure S5. Rietveld plot for the final cycle of refinement of water-guest containing Al-CAU-13 at 189 K. Left: full range of refinement; Right: fit over the 2θ range 11° to 42°.

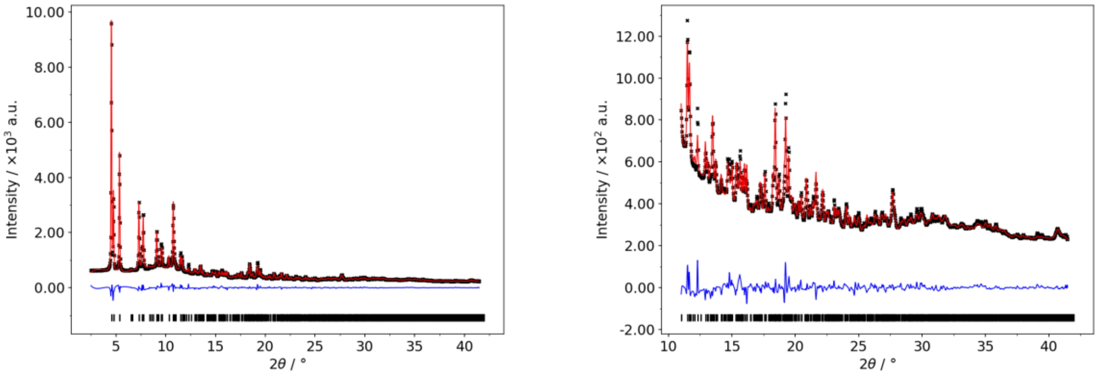

Figure S6. Rietveld plot for the final cycle of refinement of water-guest containing Al-CAU-13 at 275 K. Left: full range of refinement; Right: fit over the 2θ range 11° to 42°.

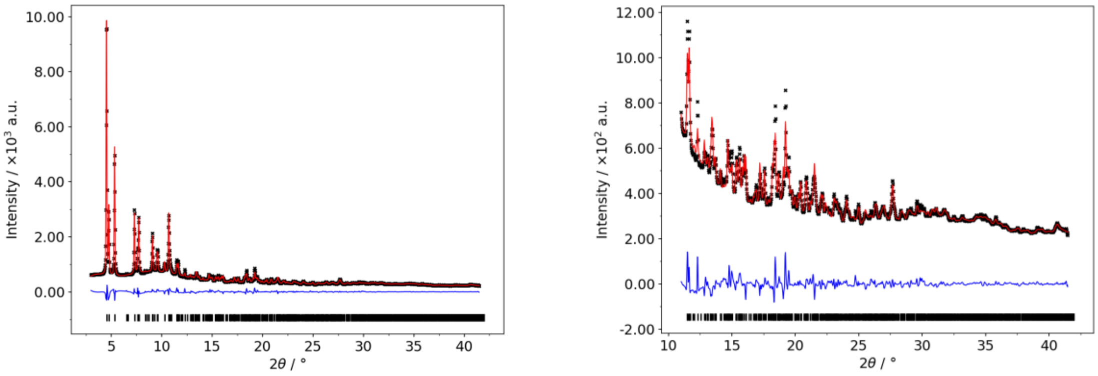

Figure S7. Rietveld plot for the final cycle of refinement of water-guest containing Al-CAU-13 at 316 K. Left: full range of refinement; Right: fit over the 2θ range 11° to 42°.

Table S2. Summary of Rietveld refinement results for guest-free Al-CAU-13 at 367 K and 500 K.

Temp. / K	367	500
Formula	$[Al(OH)(C_8H_{10}O_4)]$	$[Al(OH)(C_8H_{10}O_4)]$
Space Group	$P\bar{1}$	$P\bar{1}$
Z	2	2
a / Å	6.6252(8)	6.6207(12)
b / Å	9.8233(15)	10.027(2)
c / Å	9.4899(11)	9.5375(15)
α / °	109.229(8)	110.492(10)
β / °	107.355(12)	107.054(18)
γ / °	93.407(10)	93.335(13)
V / Å^3	548.03(14)	557.78(19)
R_{wp} / %	3.62	3.33
R_{Bragg} / %	3.01	1.87
GoF*	34.287	31.875

Figures S8 and S9 show Rietveld plots for the refinements shown in Table S2.

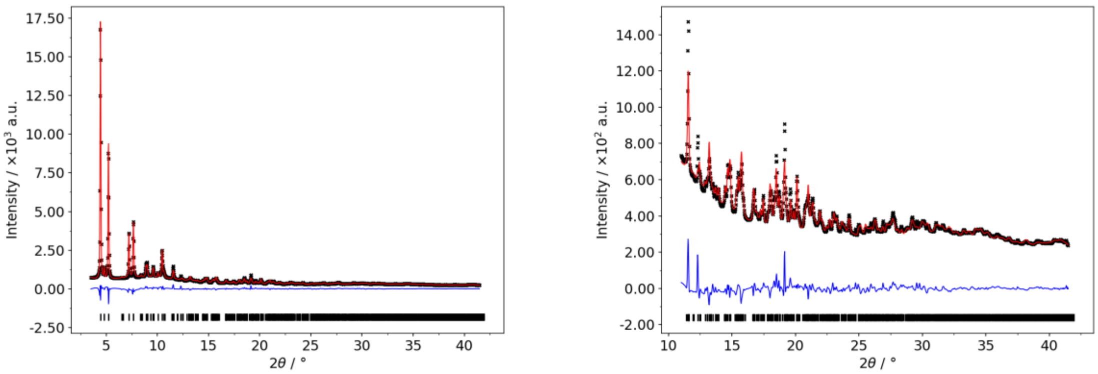

Figure S8. Rietveld plot for the final cycle of refinement of water-guest containing Al-CAU-13 at 367 K. Left: full range of refinement; Right: fit over the 2θ range 11° to 42°.

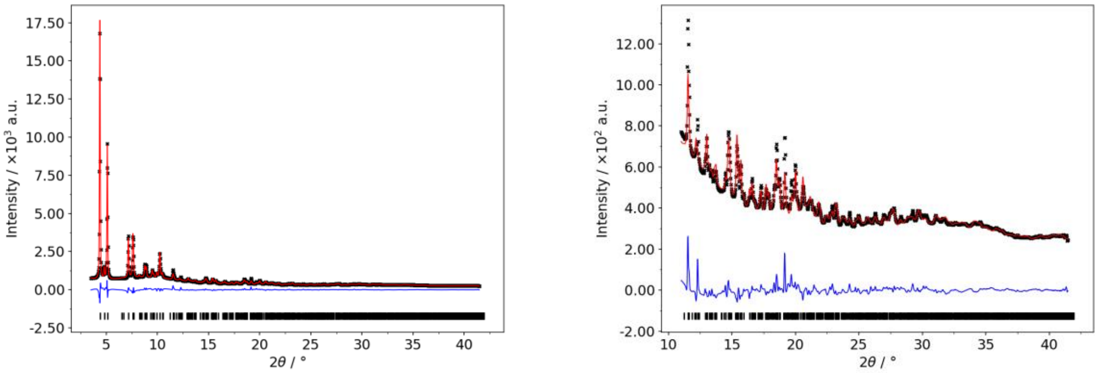

Figure S9. Rietveld plot for the final cycle of refinement of water-guest containing Al-CAU-13 at 500 K. Left: full range of refinement; Right: fit over the 2θ range 11° to 42°.

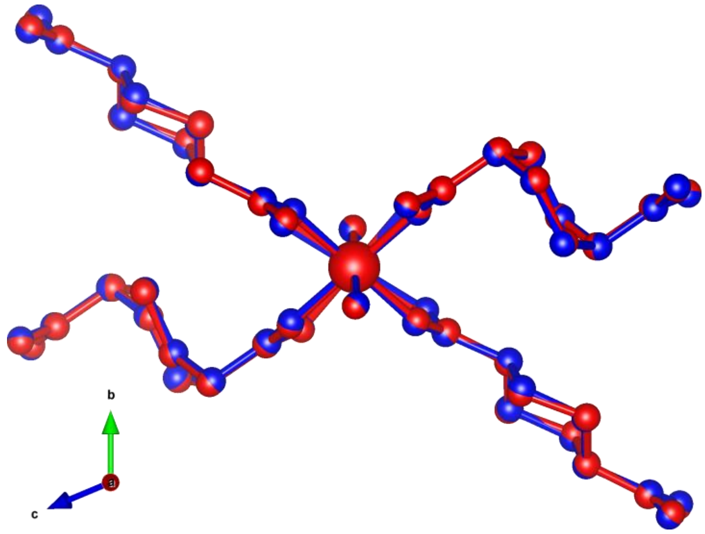

Figure S10. Structures of Al-CAU-13 at 82 K (blue) and 190 K (red) overlaid, showing differences between structures. Guest water molecules not included for clarity.

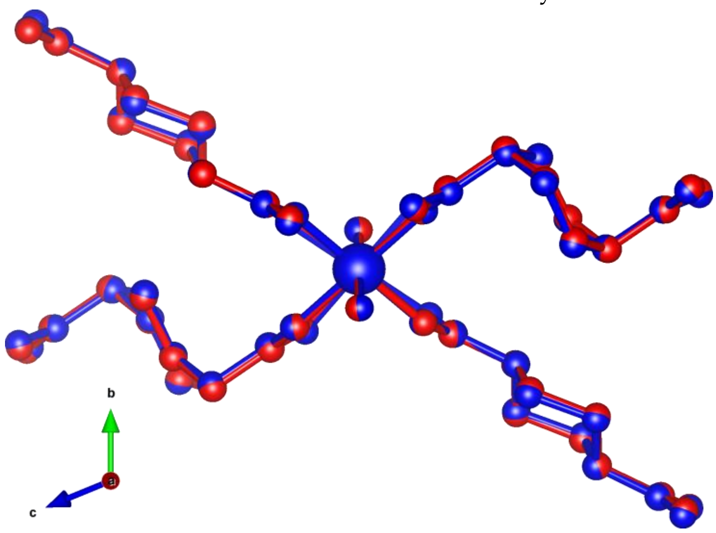

Figure S11. Structures of Al-CAU-13at 190 K (blue) and 275 K (red) overlaid, showing differences between structures. Guest water molecules not included for clarity.

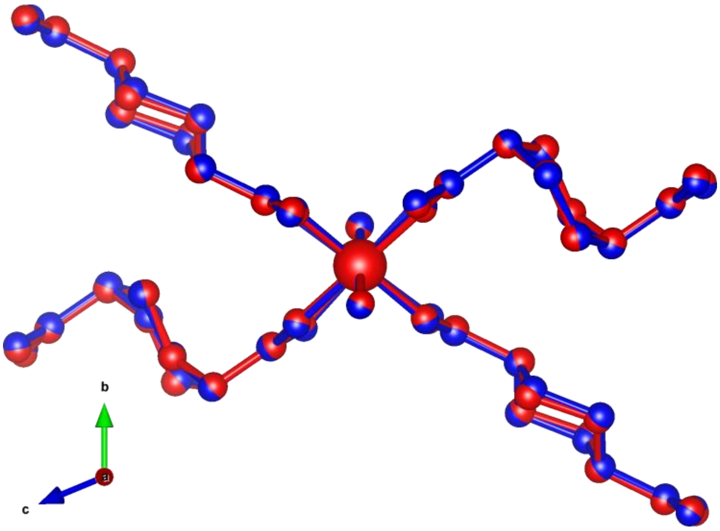

Figure S12. Structures of Al-CAU-13 at 275 K (blue) and 316 K (red) overlaid, showing differences between structures. Guest water molecules not included for clarity.

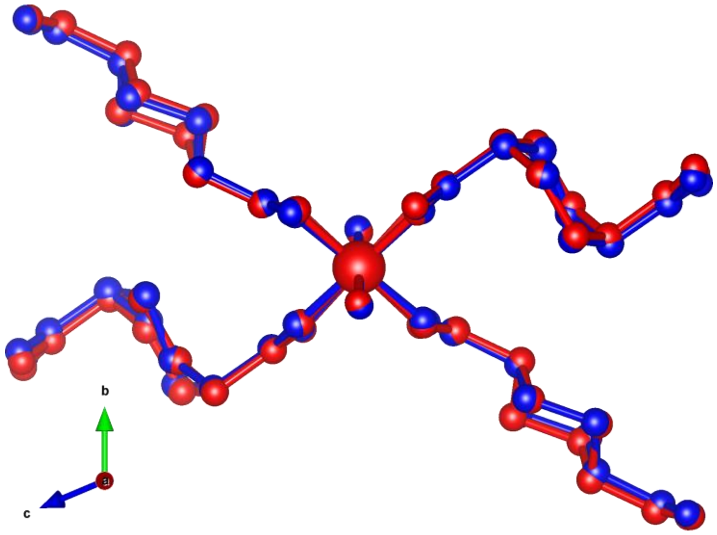

Figure S13. Structures of Al-CAU-13 at 316 K (blue) and 375 K (red) overlaid, showing changes in the structure occurring on loss of guest water molecules. Guest water molecules not included for clarity.

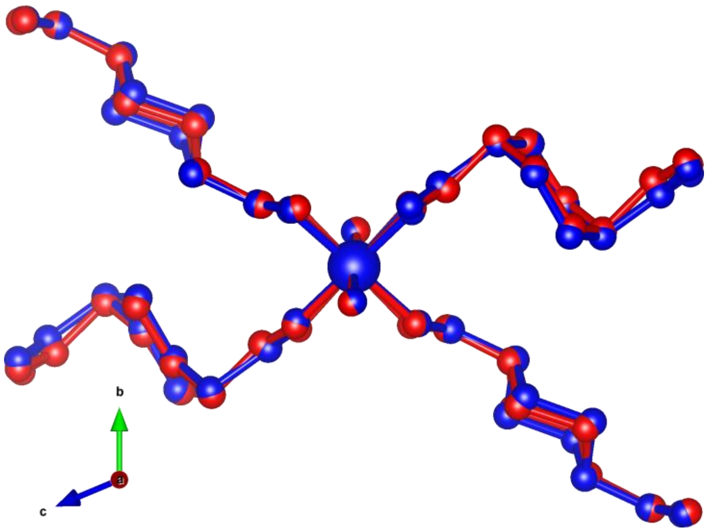

Figure S14. Structures of Al-CAU-13 at 367 K (blue) and 500 K (red) overlaid, showing differences between structures.

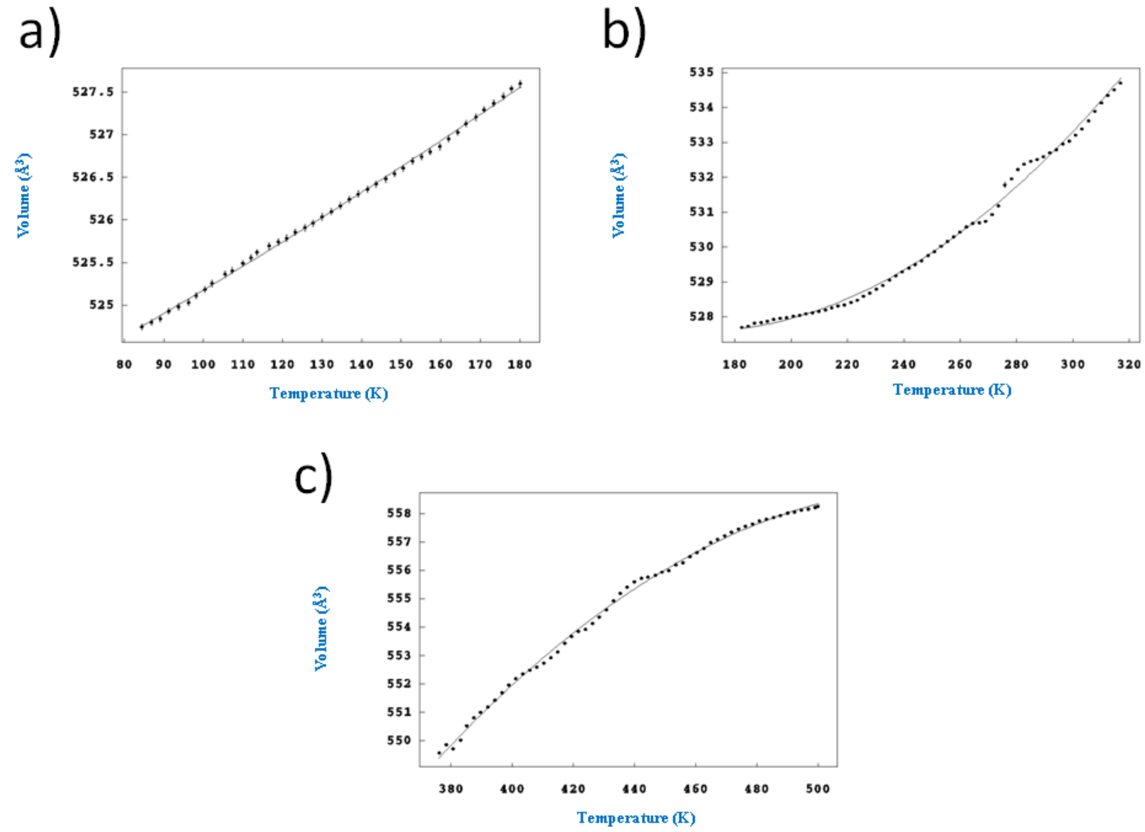

Figure S15. Evolution of the unit cell volume for Al-CAU-13 as a function of temperature for the three domains: a) low temperature linear (84-190 K), b) low temperature non-linear (190-316 K) and c) high temperature (367-500 K). Filled circles are the experimental data; determined Berman EoS plotted as a solid line.

In addition to the diffraction data reported in this work, the Equation of State (EoS) for diffraction data previously reported in Niekiel *et al.* [4] was also determined. Plots of the volume dependence of the data are given in Fig. S16 and the EoS values are given in Fig. S17 and Table S3 respectively. Data were collected in two heating processes: the first heating the sample to just below 475 K, which removed most of the water from the compound; the second, after the sample had cooled back to room temperature, up to 600 K. Data were collected on a laboratory instrument, representing a slower and potentially more completely equilibrated experiment.

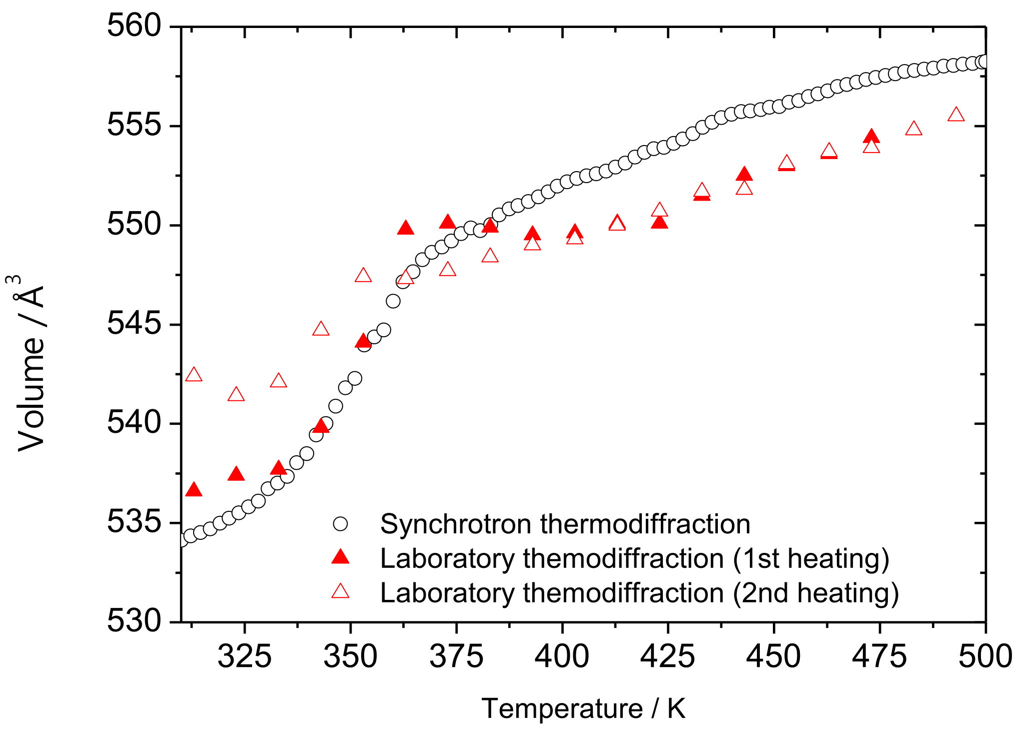

Figures S16. Comparison of unit cell volume of Al-CAU-13 over the temperature range 310 to 500 K determined from synchrotron (this study) and laboratory (data reported by Niekiel *et al.* [4]) thermodiffraction data. Niekiel *et al.* provide two sets of cell volume data: the first were obtained from an as-prepared sample of Al-CAU-13•H_2O; the second was obtained from the same sample after it had cooled from 500 K back to 303 K.

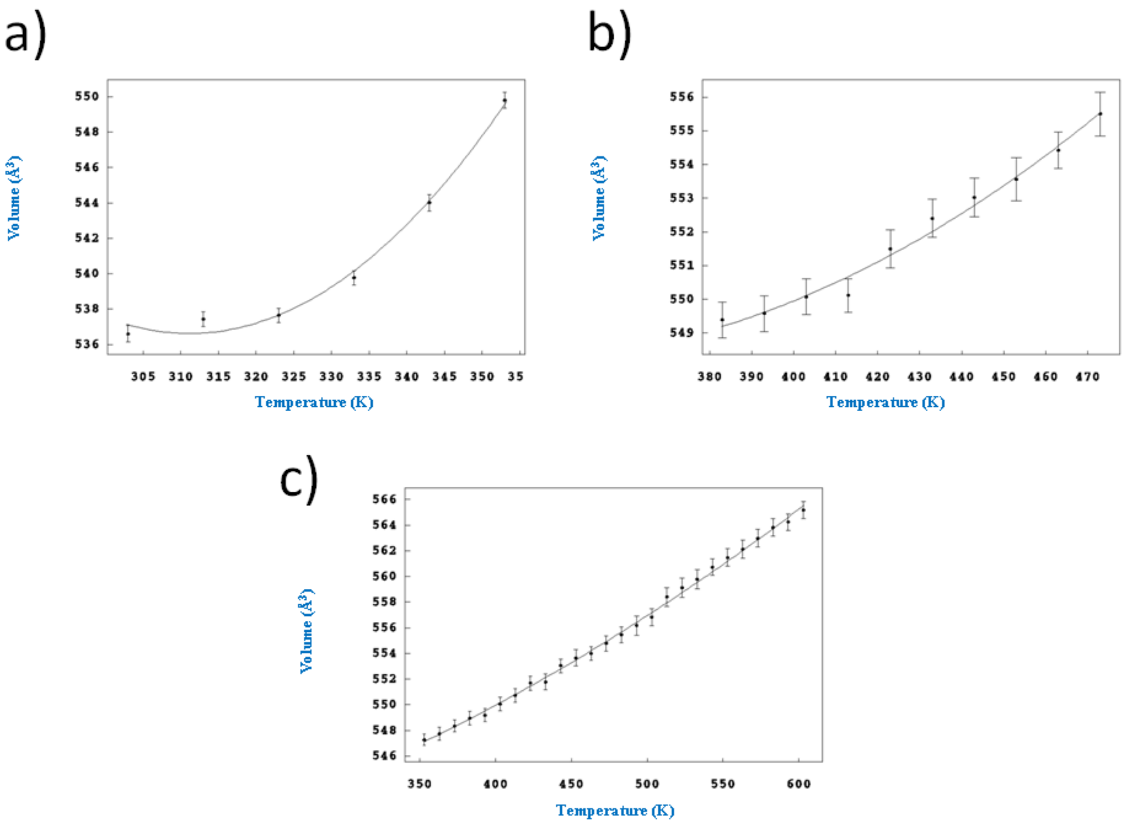

Figure S17. Evolution of the unit cell volume of Al-CAU-13 as a function of the temperature during the first and second heating experiments. The first experiment is split into two regions: (a) 305-353 K and (b) 383-473 K, with differing degrees of non-linear behavior. The first of these represents the dehydration of the compound. The second heating (c) could be fitted with a single curve over the entire region (353-673 K). Filled circles are the experimental data; determined Berman EoS plotted as a solid line.

Table S3. Parameters of the thermal Equation of State determined for the three regions plotted in Figure S17.

	**Temperature range (K)**	**V_0 (Å^3)**	**α_0 (×10^{-5} K^{-1})**	**α_1 (×10^{-8} K^{-2})**
First heating	303-353	537.1(1) at 303 K	-22.47(1)	2754.0(1)
	383-473	549.2(1) at 383 K	7.1(1)	128.7(1)
Second heating	353-673	547.1(1)	10.5(1)	23.8(1)

3. *In situ High Pressure Synchrotron X-ray Diffraction*

The following section shows plots for Pawley fits or Rietveld refinements of diffraction data measured at elevated pressure. In the plots, black crosses show measured data, the red line is the calculated diffraction pattern and the blue line is difference between measured and calculated. Expected peak positions are indicated with vertical ticks at the bottom of the plot.

3.1. Al-CAU-13•H_2O – Helium Pressure Medium

Two plots are shown: the initial loading pressure in the membrane Diamond Anvil Cell (mDAC; 0.26 GPa – Fig. S18) and the highest achieved pressure (8.09 GPa – Fig. 19). Above 10.81 GPa, one of the diamonds of the mDAC broke.

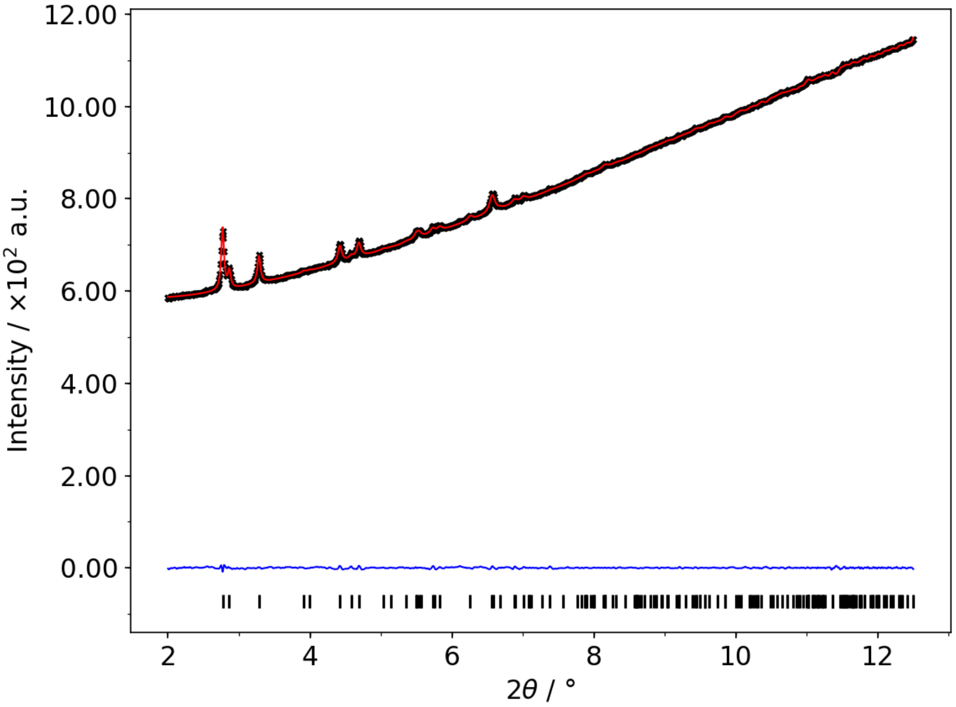

Figure S18. Plot for the Pawley fit of Al-CAU-13•H_2O at a pressure of 0.26 GPa with helium as the pressure medium (R_{wp} = 0.16 %, GoF = 0.0526).

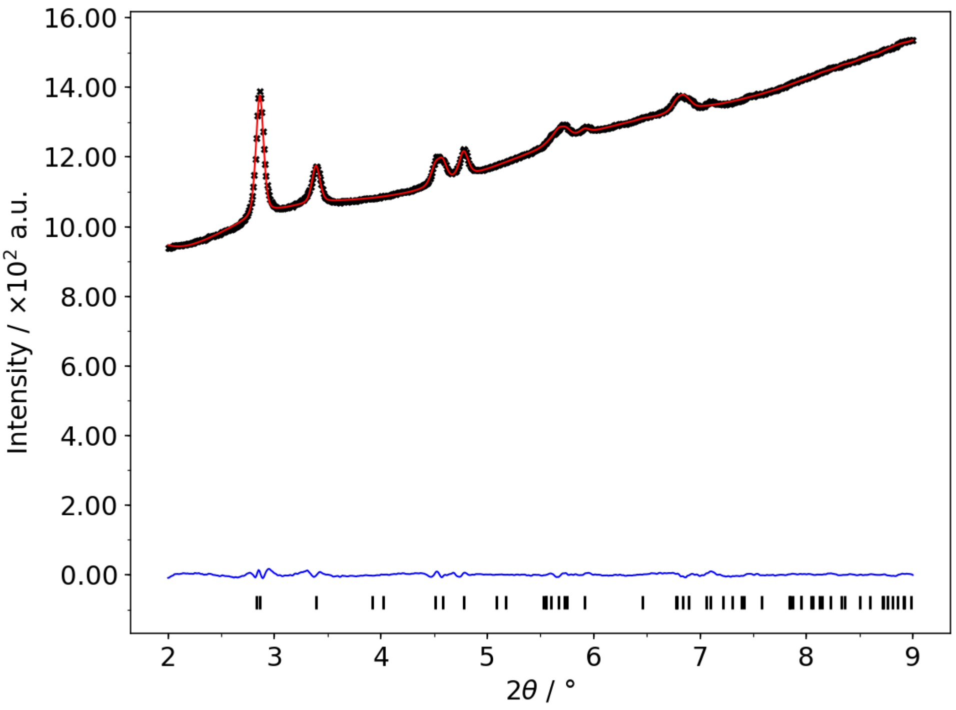

Figure S19. Plot for the Pawley fit of Al-CAU-13•H_2O at a pressure of 8.09 GPa with helium as the pressure medium (R_{wp} = 0.19 %, GoF = 0.0641).

3.2. Al-CAU-13•H_2O – Silicone Oil Pressure Medium

AP100 silicone oil was used as the pressure medium. Three plots are shown: the initial loading pressure in the mDAC (0.42 GPa – Fig. S20), the refinement at the end of the first phase of (elastic) compression (1.75 GPa – Fig. S21) and the refinement at the highest pressure achieved in the experiment (10.80 GPa – Fig. S22). It should be noted that in this final refinement, the peak positions were too unreliable and therefore not used for determining the equation of state. Furthermore, with increasing pressure the cell volumes generally became more unreliable (larger ESDs) and were therefore given a lower weighting in the least squares fitting of the EoS.

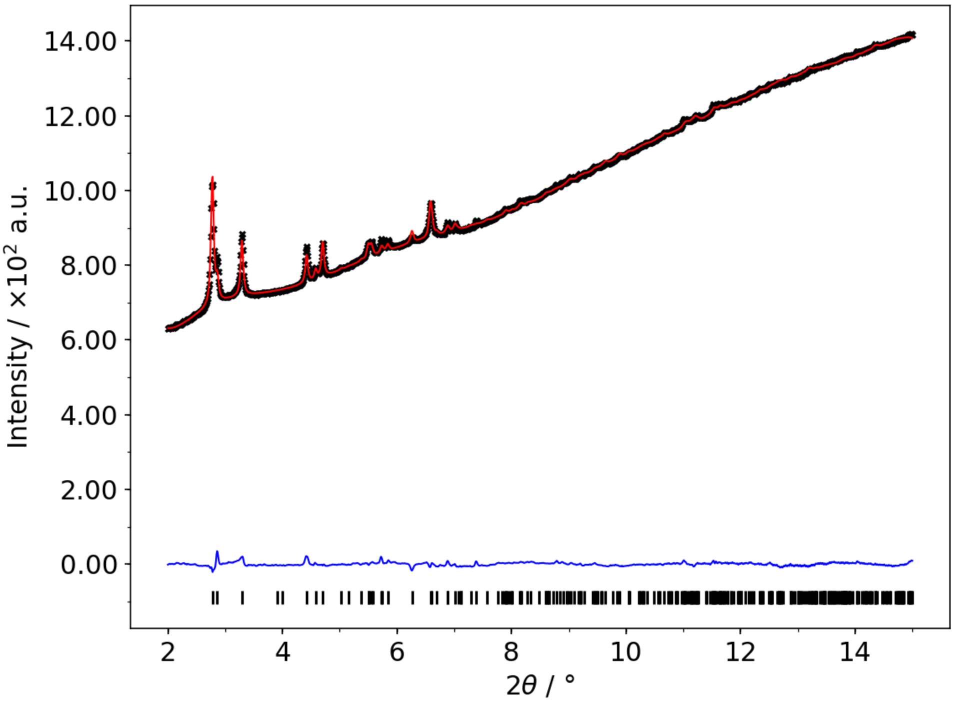

Figure S20. Rietveld plot for the refinement of of Al-CAU-13•H_2O at a pressure of 0.42 GPa with silicone oil as the pressure medium (R_{wp} = 0.47 %, GoF = 0.1518).

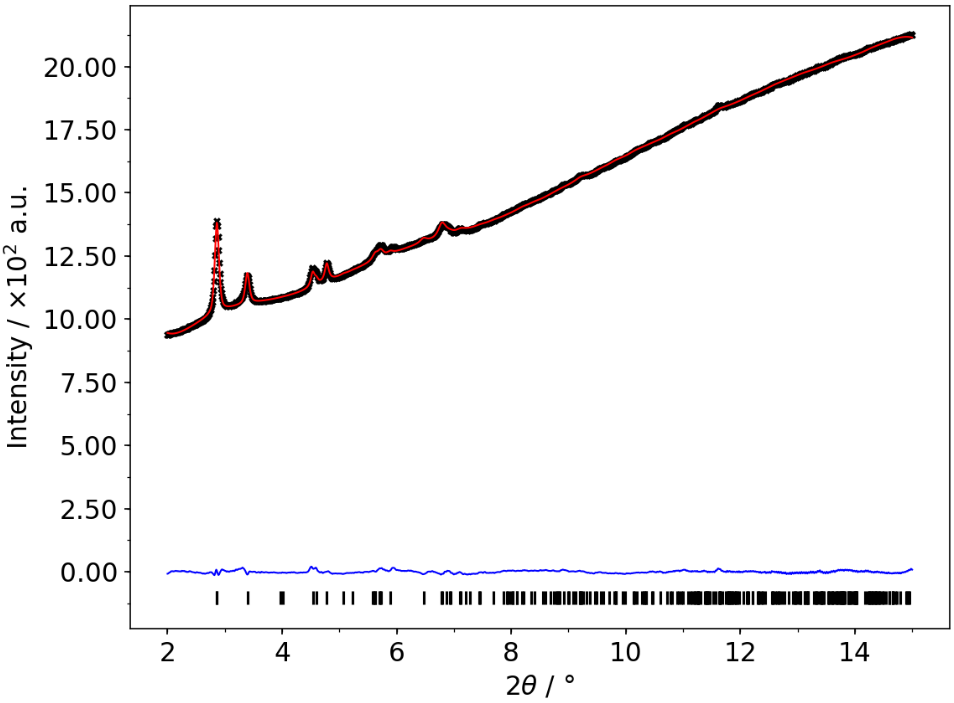

Figure S21. Rietveld plot for the refinement of of Al-CAU-13•H_2O at a pressure of 1.75 GPa with silicone oil as the pressure medium (R_{wp} = 0.35 %, GoF = 0.1376).

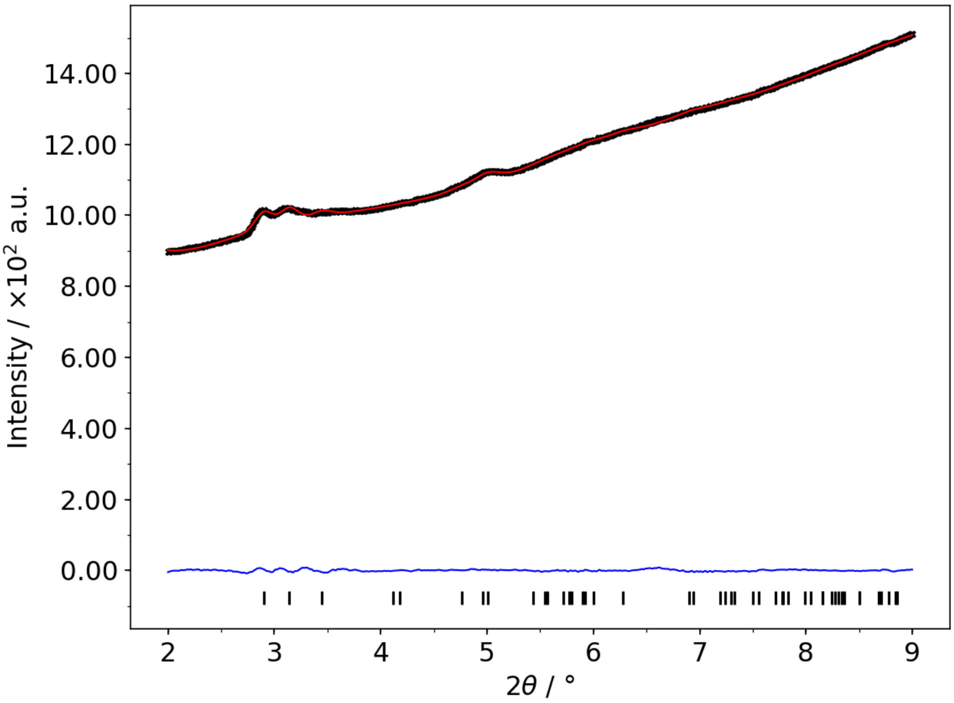

Figure S22. Plot for the Pawley fit of Al-CAU-13•H_2O at a pressure of 10.80 GPa with silicone oil as the pressure medium (R_{wp} = 0.23 %, GoF = 0.0799). N.B. A smaller 2θ range (compared to the lower pressure measurements) was used for the refinement to ensure it remained stable.

3.3. Al-CAU-13@Tet – Silicone Oil Pressure Medium

For the study of Al-CAU-13@Tet, AP100 silicone oil was again used. The structure remains orthorhombic across the entire pressure range, from 0.27 GPa (i.e. only the pressure of the loading – Fig. S23) to 4.19 GPa (Fig. S24).

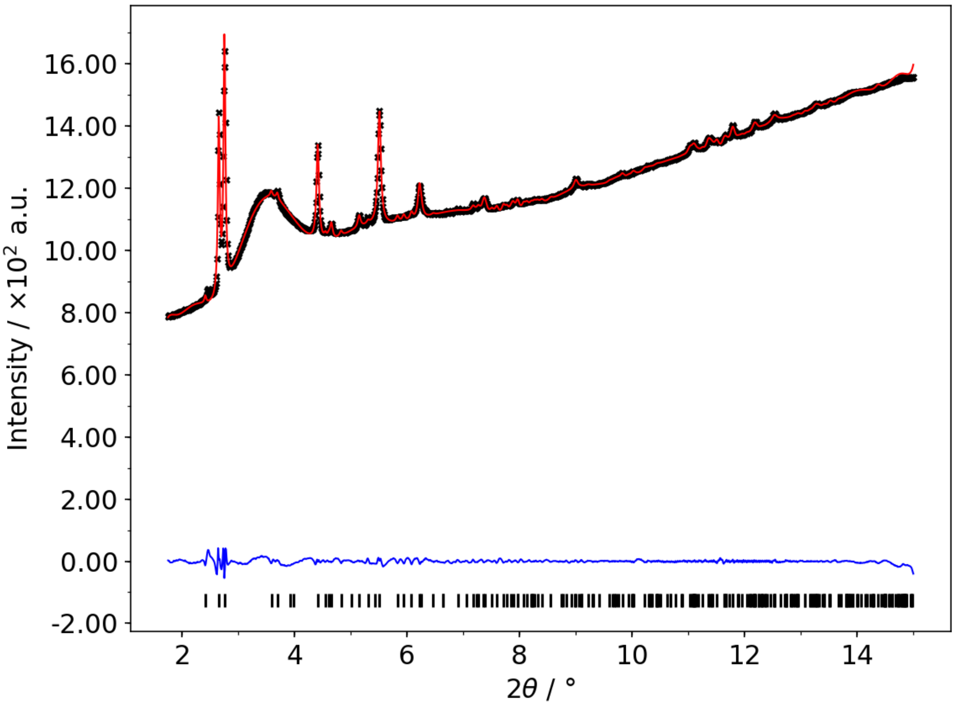

Figure S23. Plot for the Pawley fit of Al-CAU-13@Tet at a pressure of 0.27 GPa with silicone oil as the pressure medium (R_{wp} = 0.56 %, GoF = 0.2193).

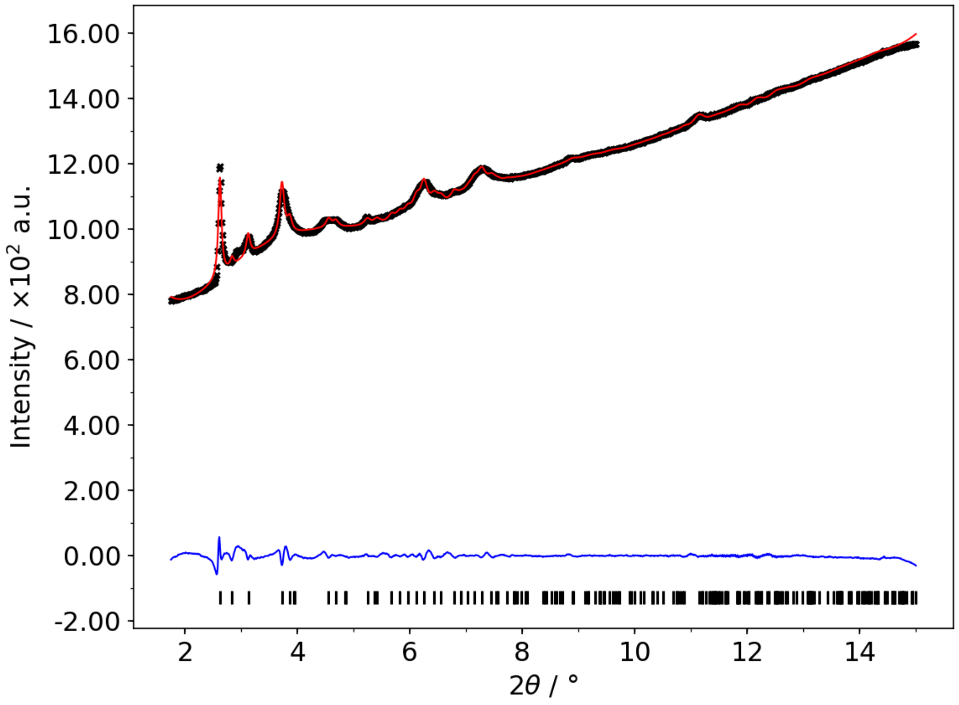

Figure S24. Plot for the Pawley fit of Al-CAU-13@Tet at a pressure of 4.19 GPa with silicone oil as the pressure medium (R_{wp} = 0.68 %, GoF = 0.2569).

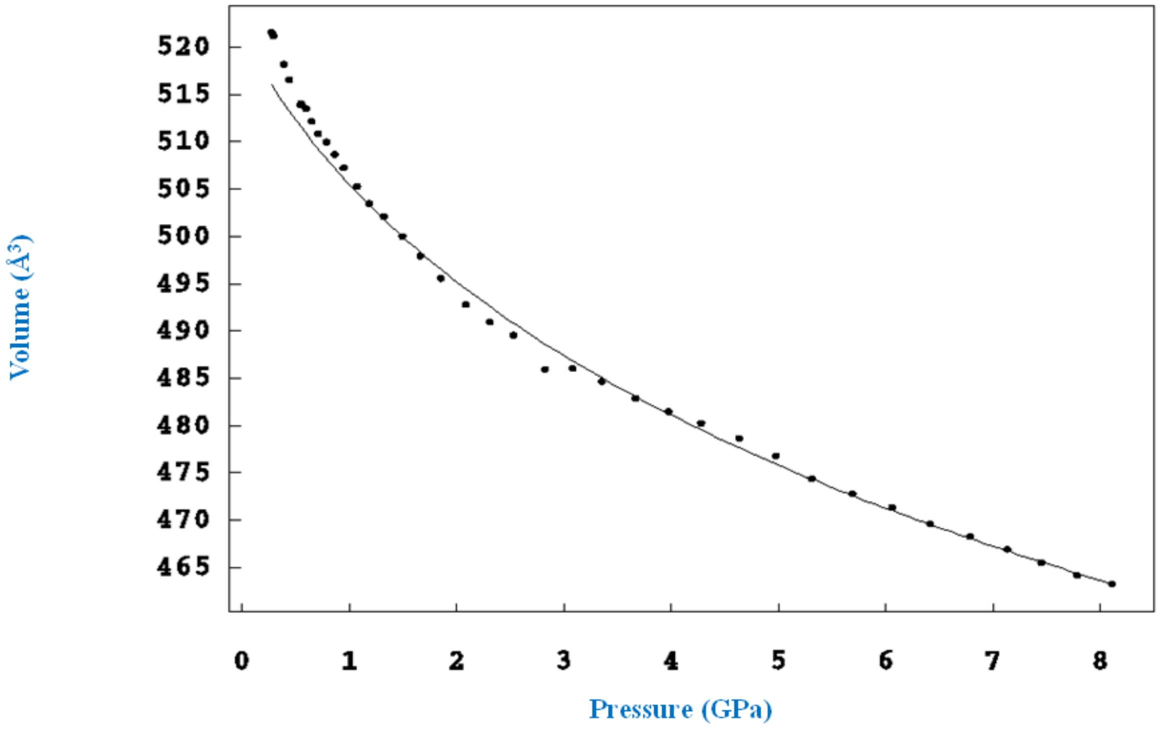

Figure S25. Evolution of the unit cell volume for the Al-CAU-13•H_2O in He over the pressure range 0.28-8.11 GPa (points) with the curve fit for the Vinet EoS.

(a)

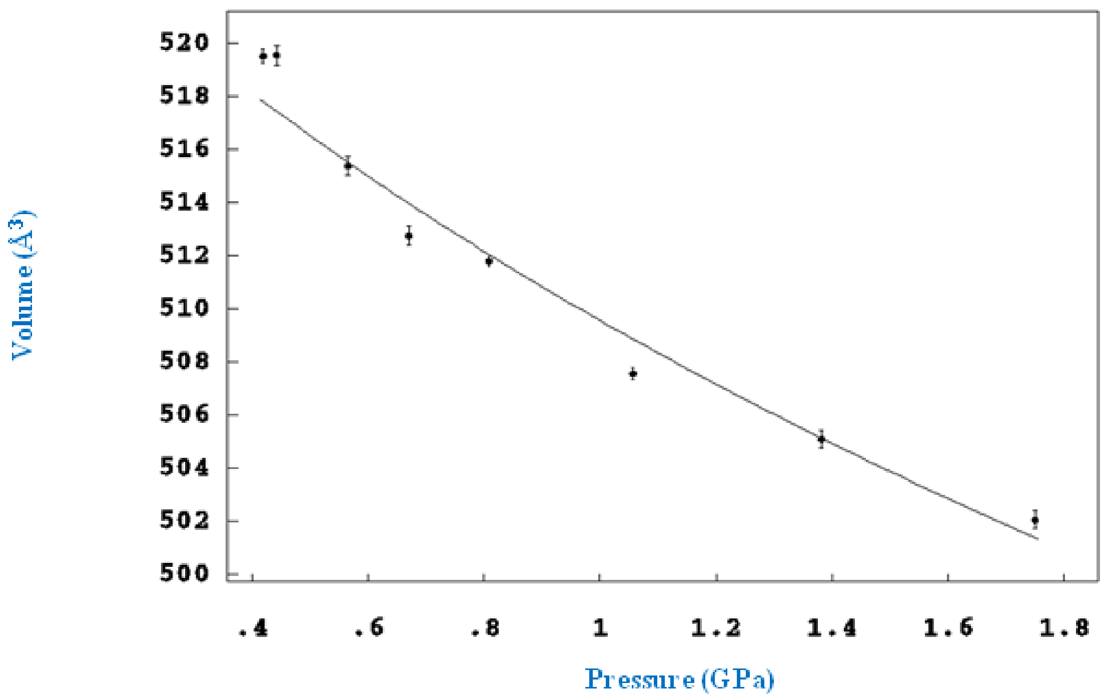

(b)

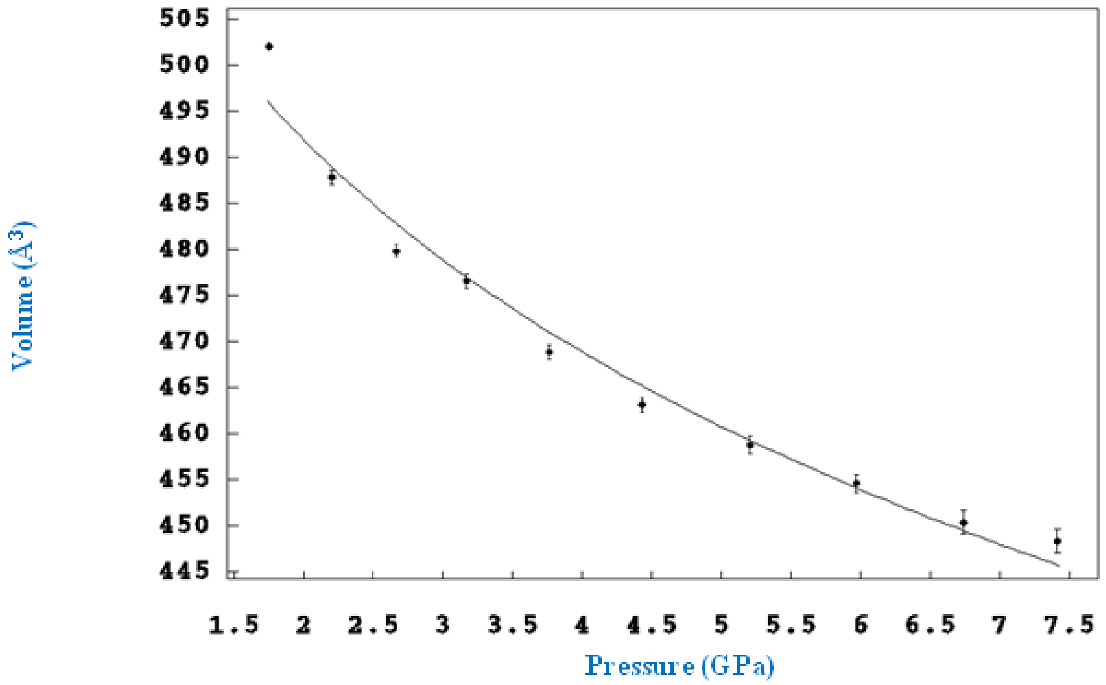

Figure S26. Evolution of the unit cell volume for the Al-CAU-13•H_2O in silicone oil over the pressure ranges (a) 0.42-1.75 GPa and (b) 1.75-7.41 GPa (points). The curve is the fit for the Vinet EoS.

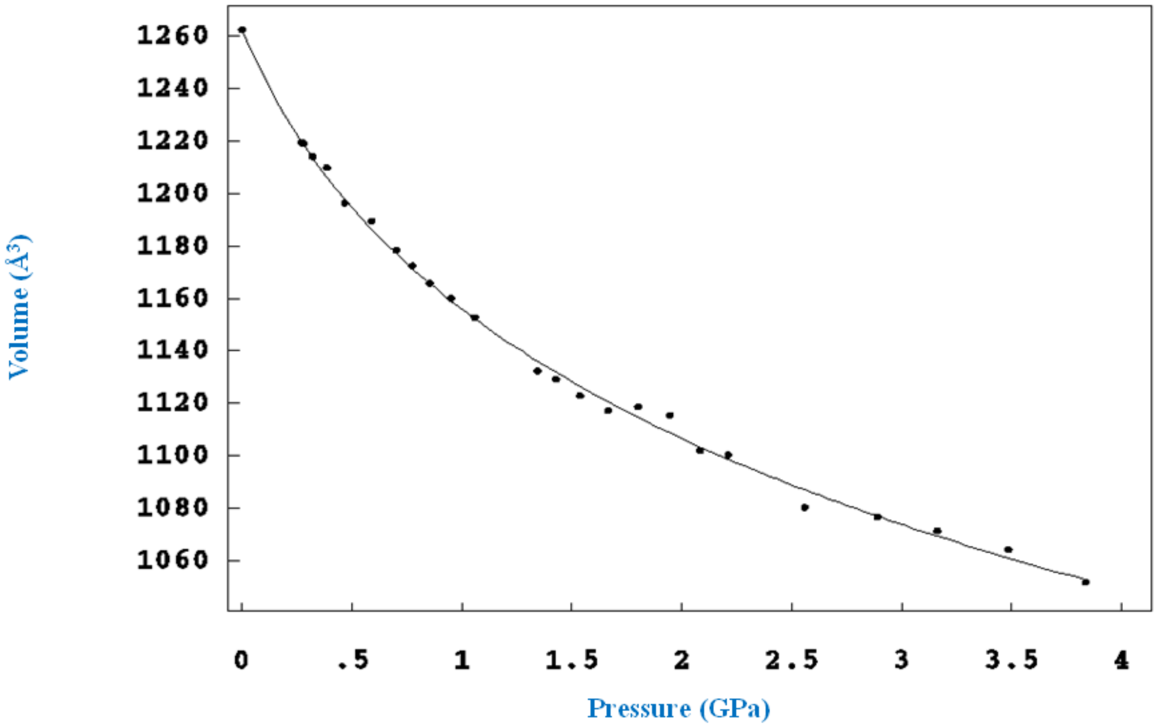

Figure S27. Evolution of the unit cell volume for the Al-CAU-13@Tet in silicone oil over the pressure range 0.27-3.84 GPa (points) with the curve fit for the Vinet EoS.

4. *Equations of State Used for Fits of Thermal & Mechanical Data*

The following terms are used in the equations of state:

V – cell volume; V_{0x} – cell volume under reference conditions (x indicates Temperature or pressure)

T – temperature; T_{Ref} – reference temperature

α_x – thermal expansion coefficient (x denotes the order of the coefficient)

p – pressure

K_0 – Bulk modulus; K′ – isothermal first derivative of bulk modulus with respect to pressure.

4.1. Berman Equation of State

$$V_T = V_{0T}(1 + \alpha_0(T - T_{Ref}) + \frac{1}{2}\alpha_1(T - T_{Ref})^2)$$

After Berman [2].

4.2. Vinet Equation of State

$$p = 3K_0 \left[\frac{1 - X}{X^2}\right] \exp\left(\eta(1 - X)\right)$$

where,

$$X = \left(\frac{V}{V_{0p}}\right)^{\frac{1}{3}}$$

$$\eta = \frac{3}{2}(K' - 1)$$

After Vinet *et al.* [3].

5. References

1. Reinsch, H.; Benecke, J.; Etter, M.; Heidenreich, N.; Stock, N. Combined in- and ex situ studies of pyrazine adsorption into the aliphatic MOF Al-CAU-13: structures, dynamics and correlations. *Dalton Trans.* **2017**, *46*, 1397–1405, doi:10.1039/C6DT03998G.
2. Berman, R.G. Internally-consistent thermodynamic data for minerals in the system Na_2O-K_2O-CaO-MgO-FeO-Fe_2O_3-Al_2O_3-SiO_2-TiO_2-H_2O-CO_2. *Journal of Petrology* **1988**, *29*, 445–522.
3. Vinet, P.; Ferrante, J.; Rose, J.H.; Smith, J.R. Compressibility of solids. *J. Geophys. Res.* **1987**, *92*, 9319, doi:10.1029/JB092iB09p09319.
4. Niekiel, F.; Lannoeye, J.; Reinsch, H.; Munn, A.S.; Heerwig, A.; Zizak, I.; Kaskel, S.; Walton, R.I.; de Vos, D.; Llewellyn, P.; *et al*. Conformation-Controlled Sorption Properties and Breathing of the Aliphatic Al-MOF [Al(OH)(CDC)]. *Inorg. Chem.* **2014**, *53*, 4610–4620, doi:10.1021/ic500288w.
